# Comparing Survival Extrapolation within All-Cause and Relative Survival Frameworks by Standard Parametric Models and Flexible Parametric Spline Models Using the Swedish Cancer Registry

**DOI:** 10.1177/0272989X241227230

**Published:** 2024-02-05

**Authors:** Enoch Yi-Tung Chen, Yuliya Leontyeva, Chia-Ni Lin, Jung-Der Wang, Mark S. Clements, Paul W. Dickman

**Affiliations:** Department of Medical Epidemiology and Biostatistics, Karolinska Institutet, Stockholm, Sweden; Department of Medical Epidemiology and Biostatistics, Karolinska Institutet, Stockholm, Sweden; Department of Public Health, College of Medicine, National Cheng Kung University, Tainan, Taiwan; Department of Public Health, College of Medicine, National Cheng Kung University, Tainan, Taiwan; Department of Occupational and Environmental Medicine, National Cheng Kung University Hospital, College of Medicine, National Cheng Kung University, Tainan, Taiwan; Department of Medical Epidemiology and Biostatistics, Karolinska Institutet, Stockholm, Sweden; Department of Medical Epidemiology and Biostatistics, Karolinska Institutet, Stockholm, Sweden

**Keywords:** survival extrapolation, flexible parametric models, spline models, restricted mean survival time, life expectancy, relative survival, cancer registry, cost-effectiveness analysis

## Abstract

**Background:**

In health technology assessment, restricted mean survival time and life expectancy are commonly evaluated. Parametric models are typically used for extrapolation. Spline models using a relative survival framework have been shown to estimate life expectancy of cancer patients more reliably; however, more research is needed to assess spline models using an all-cause survival framework and standard parametric models using a relative survival framework.

**Aim:**

To assess survival extrapolation using standard parametric models and spline models within relative survival and all-cause survival frameworks.

**Methods:**

From the Swedish Cancer Registry, we identified patients diagnosed with 5 types of cancer (colon, breast, melanoma, prostate, and chronic myeloid leukemia) between 1981 and 1990 with follow-up until 2020. Patients were categorized into 15 cancer cohorts by cancer and age group (18–59, 60–69, and 70–99 y). We right-censored the follow-up at 2, 3, 5, and 10 y and fitted the parametric models within an all-cause and a relative survival framework to extrapolate to 10 y and lifetime in comparison with the observed Kaplan-Meier survival estimates. All cohorts were modeled with 6 standard parametric models (exponential, Weibull, Gompertz, log-logistic, log-normal, and generalized gamma) and 3 spline models (on hazard, odds, and normal scales).

**Results:**

For predicting 10-y survival, spline models generally performed better than standard parametric models. However, using an all-cause or a relative survival framework did not show any distinct difference. For lifetime survival, extrapolating from a relative survival framework agreed better with the observed survival, particularly using spline models.

**Conclusions:**

For extrapolation to 10 y, we recommend spline models. For extrapolation to lifetime, we suggest extrapolating in a relative survival framework, especially using spline models.

**Highlights:**

- For survival extrapolation to 10 y, spline models generally performed better than standard parametric models did. However, using an all-cause or a relative survival framework showed no distinct difference under the same parametric model.
- Survival extrapolation to lifetime within a relative survival framework agreed well with the observed data, especially using spline models.
- Extrapolating parametric models within an all-cause survival framework may overestimate survival proportions at lifetime; models for the relative survival approach may underestimate instead.

## Background

Restricted mean survival time (RMST) or life expectancy (LE) are common survival outcomes evaluated in health technology assessment (HTA). Moreover, estimation of quality-adjusted life-years is often based on calculating the mean survival times and utility values. Interest typically lies in the experience of patients who are recently diagnosed or participating in ongoing clinical trials. Hence, survival data are usually still immature (right censored). Extrapolation is then required to predict survival beyond follow-up to obtain estimates for RMST or LE.^1^ In health economic evaluations, survival extrapolation is typically carried out by fitting the observed survival data with parametric models and subsequently predicting survival probabilities based on estimated model parameters.^2–4^ However, the parametric distribution may fit the observed data well but generate poor extrapolation because it may not be sufficiently flexible to capture the underlying shape of a hazard function in the long run.^5,6^ An unsuitable choice of the model may lead to biased extrapolations, which may eventually result in inaccurate cost-effectiveness analysis results in HTA.^2^

Previous recommendations from National Institute for Health and Care Excellence (NICE)’s Technical Support Document 14 suggested that 6 standard parametric models (SPMs)—exponential, Weibull, Gompertz, log-logistic, log-normal, and generalized gamma models—should be considered as selecting appropriate models for survival extrapolation.^3^ More recent guidance from NICE’s Technical Support Document 21 published in 2021 provided general recommendations on using flexible parametric models incorporating restricted cubic splines.^5^ In this study, we refer to flexible parametric models^7^ as spline models. Recent studies have compared multiple statistical models, including SPMs, spline models, cure, mixture, and landmark models for survival extrapolation for patients treated by cancer immunotherapies. However, the results were compared only with the observed survival of 3 y^8^ and 5 y.^9^ Gray et al.^10^ applied SPMs and spline models for survival extrapolation and further compared their performance using long follow-up cancer registry survival data. They suggested spline models should be routinely applied for extrapolating cancer survival data. However, they evaluated only models in an all-cause survival framework (ASF), that is, extrapolation of all-cause hazard (mortality) models, for 10-y survival outcomes instead of longer survival outcomes, such as LE.^10^

A review by Jackson et al.^11^ summarized survival extrapolation approaches integrating external data, including survival extrapolation within a relative survival framework (RSF),^5,6^ also referred as an excess hazard (mortality) framework.^12^ Relative survival, R(t) , is defined as the all-cause survival of the patients, S(t) , divided by the expected survival of a comparable population free from the disease under study, $S^{*}\left(t\right)$ , written as

$$R\left(t\right)=\frac{S\left(t\right)}{S^{*}\left(t\right)},$$

where t is time since diagnosis.^13^ The hazard analogue of relative survival is excess hazard. The all-cause hazard, h(t), is the sum of the expected hazard, $h^{*}\left(t\right)$ , and the excess hazard ,λ(t) , shown as

$$h\left(t\right)=h^{*}\left(t\right)+\lambda \left(t\right).$$

To extrapolate survival within an RSF, one needs to extrapolate the relative survival and the expected survival, that is, decompose the all-cause hazard into the expected hazard and the excess hazard, and extrapolate them separately. Extrapolation of expected survival can be carried out by projecting the expected hazard, in other words, predicting future general population mortality rates (GPMRs).^6^ Extrapolation of relative survival can be done by modeling and predicting the excess hazard.^14^ Afterward, one may use the interrelationship between the relative survival, all-cause survival, and expected survival to obtain the extrapolated all-cause survival.

For most cancers, the excess hazard, that is, the mortality attributed to cancer, decreases with time and remains low or reaches zero for a longer period.^15^ This characteristic potentially favors fitting a parametric model to capture the underlying excess hazard function and extrapolate it. Furthermore, the expected hazard (from GPMRs) may explain a substantial part of the all-cause hazard in the long-run.^16,17^ Therefore, extrapolation within an RSF can be reasonable for projecting long-term hazards. Andersson et al.^6^ showed that extrapolating lifetime survival more reliably using spline models within an RSF. Their main focus was on estimating loss in LE rather than short-term survival outcomes, for example, 10-year RMST.^6^

One goal of extrapolation is to apply appropriate parametric models for predicting survival in randomized clinical trials. Clinical trial survival data usually do not have complete follow-up of the study population, leading to unknown long-term survival outcomes. An alternative to assessing the performance of survival extrapolation is to adopt observational data, for example, population-based cancer registers. The patterns of hazard functions across cancer sites and ages may be largely heterogeneous. Therefore, they offer a variety of survival functions for evaluating extrapolation approaches. Furthermore, cancer registers provide longer follow-up of known survival outcomes, which enables us to compare with survival extrapolations.^10^ The Swedish Cancer Registry was established in 1958, and its completeness is greater than 96%.^18^ All healthcare workers in Sweden are required to register all incident cancer cases with information such as age, sex, date of diagnosis, type of diagnosis, date of emigration, and so on. Accordingly, this study used these data from the real-world cancer registry to evaluate survival extrapolation methods.

This research aims to investigate the performance of survival extrapolations within an ASF and an RSF using SPMs and spline models for 10-y and lifetime survival outcomes using the Swedish Cancer Registry. To allow for a variety of hazard function shapes, we included 5 cancer sites: 2 sex-specific cancer sites (breast and prostate cancer) and other 3 non–sex-specific ones (colon cancer, melanoma, and chronic myeloid leukemia). Furthermore, we limited the follow-up time of the survival data and extrapolated by parametric models in an ASF and an RSF to compare with the observed Kaplan–Meier (K-M) survival estimates.

## Methods

### Study Population

This study used the Swedish Cancer Registry to identify patients diagnosed with colon cancer (International Classification of Diseases version 7 code 153.x), breast cancer (code 170.x), malignant melanoma (code 190.x), prostate cancer (code 177.x), and chronic myeloid leukemia (CML) (code 205.1) at 18 to 99 y old in Sweden during 1981–1990, with follow-up until December 31, 2020. Information regarding the date of death was obtained from the Cause of Death Register via the linkage with each patient’s Swedish unique registration number. Patients who emigrated were classified as censored on the date of first emigration. For individuals with multiple primary recorded tumors of the same type, we included only the first diagnosis. Patients diagnosed at autopsy were excluded. For breast cancer, males (<1%) were excluded from the analyses.

### Survival Models

To align with the study by Gray et al.,^10^ we classified the patients into 3 separate age groups (18–59, 60–69, 70–99 y) to produce a total of 15 cancer cohorts. Each cancer cohort was fitted by the SPMs and spline models within an ASF and an RSF.^14,19,20^ The SPMs included a total of 6 models: exponential, Weibull, Gompertz, log-logistic, log-normal, and generalized gamma.^21^ The spline models were fitted with restricted cubic splines on 3 different scales: log cumulative hazard, log cumulative odds, and normal equivalent deviate (probit) scale with (m+1) degrees of freedom (df).^19^ For flexible parametric spline models, (m+1) df indicates that m internal knots and 2 boundary knots are applied for the restricted cubic spline function used for the baseline hazard function,^19^ and the suggested knot locations are based on the centiles of the distribution of uncensored log event times.^22^ To maintain the consistency across models, all the spline models were modelled with 5 df, that is, 4 internal knots.

For survival extrapolation within an RSF, the extrapolation of the expected survival is also required. We calculated the expected survival function with the Ederer I estimator,^13^ which can be interpreted as the unbiased survival the patients would have had if they had not been diagnosed with the disease. The Ederer I method calculates the expected survival for the patient population from the GPMRs, stratified by age, sex, and calendar year.^23^ We retrieved the GPMRs of Sweden until 2020 from the Human Mortality Database.^24^ For mortality beyond 2020, we assumed that they are equal to 2020. Detailed step-by-step instructions on survival extrapolation within an RSF can be found in the tutorial by Sweeting et al.^12^

### Evaluating Survival Extrapolation

To compare with the extrapolations, we used the K-M survival estimates of 40-y follow-up data as the observed survival outcomes. We presented the K-M survival curves with 95% confidence intervals (CIs), and the numbers at risk of each cancer cohort at selected time points. To evaluate predicted 10-y survival outcomes (10-y RMST and survival proportions at 10 y), the follow-up time for all surviving patients was right-censored at 2, 3, and 5 y and extrapolated by the parametric models to 10 y. For lifetime (or 40-y) survival outcomes (LE or 40-y RMST and survival proportions at lifetime or 40 y), the follow-up was right-censored at 2, 3, 5, and 10 y and extrapolated to lifetime (or 40 y). The LE was calculated by integrating the area under the survival curve. Namely, to obtain LE, we require the patients to have been followed until the survival has already reached zero. However, the survival proportions of some younger cancer patients were not yet zero by 40 y, so their 40-y RMST was assessed instead of LE.

We presented boxplots summarizing the differences (extrapolated minus observed) across 15 cancer cohorts (5 cancer sites by 3 age groups). These comparisons were made across 9 models, including 6 SPMs and 3 spline models, withinin either an ASF or an RSF. Varying follow-up time points, including 2, 3, 5, or 10 y, were used for extrapolation to 10 y or lifetime (40 y). Box plots show the median, first quartile, third quartile, interquartile range, and outliers for the difference. Furthermore, to evaluate the precision of the extrapolations, we presented the frequency of models in a table that predicted a difference less than or equivalent to 0.1 y of 10-y RMST, 1% survival proportion at 10 y, 1 y from the observed LE or 40-y RMST, and 1% at lifetime or 40 y, across all groups by time used for extrapolation and survival framework, where the cut points were selected subjectively. Larger numbers of frequencies imply better extrapolation performance.

For visual assessment on the extrapolation performance, we also included all the survival and hazard functions in the Supplementary Materials (Supplementary Appendices C–F). The observed quantities were from the full follow-up of 10 y or lifetime (or 40 y). The observed survival functions were from the K-M survival estimators. The observed all-cause (or excess) hazard functions were fitted by spline models^7^ on log cumulative (excess) hazard scales, with 5 df for baseline effects. The observed expected hazard functions were obtained from the observed all-cause hazard functions subtracted by the observed excess hazard functions.

For presenting the observed hazard function, we justify our choice of using spline models over nonparametric kernel-smoothed estimators based on the following reasons: the sensitivity of the kernel-smoothing method to bandwidth and the assumption of constant hazard over the bandwidth.^25^ Notably, the hazard function may change rapidly at the boundaries, which makes the kernel smoothing less suitable. This characteristic may introduce bias especially after long follow-up, where only few patients are at risk. In addition, it is more common to show parametric excess hazard functions in practice instead of nonparametric estimates. Therefore, our preference leans toward employing spline models to present smooth functions of the observed all-cause and excess hazards over the full follow-up duration. It is worth noting that the hazard functions primarily serve for visual assessment on various shapes over time at Appendices C to F. However, the main results involve only comparing the extrapolated and the observed K-M survival curves, which provide a more illustrative insight into how well the model fits the survival data.

### Statistical Software

All statistical analyses were performed in Stata version 17.0 (Statacorp, College Station, TX, USA). All SPMs and spline models were fitted and postestimated by the Stata commands *merlin*^20^ and *stpm2*^19^ separately.

## Results

### Baseline Characteristics and Observed Survival Outcomes

**Figure 1:**
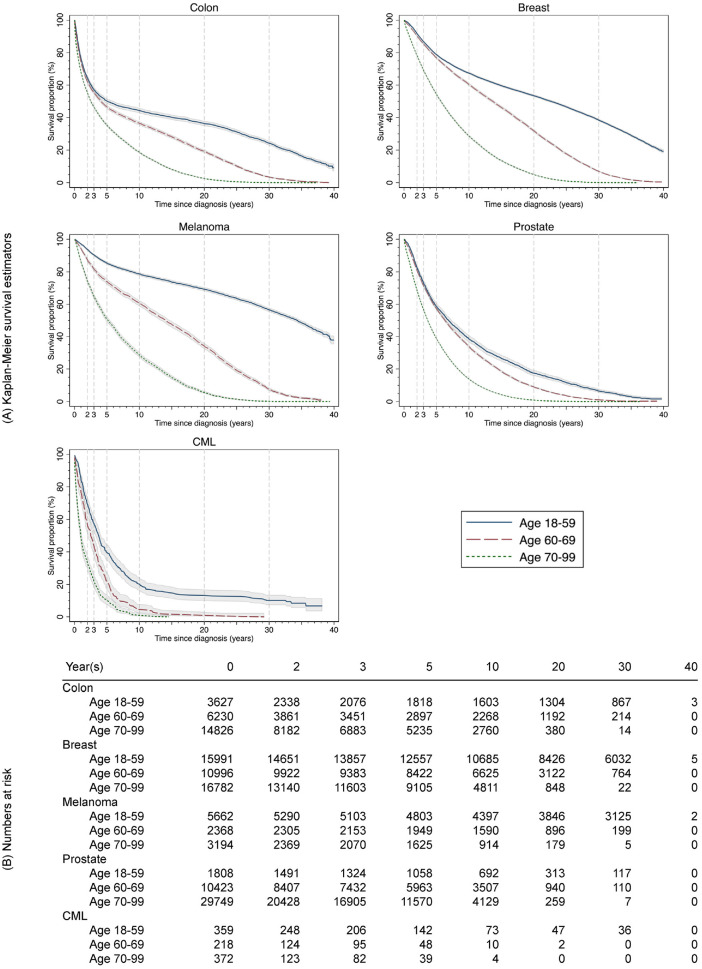
(A) Kaplan-Meier survival estimates (lines) with 95% confidence intervals (shaded areas) and (B) numbers at risk for 15 cancer cohorts, diagnosis in Sweden during 1981-1990 with follow-up until 2020. Vertical dashed lines on plot (A) indicate time points at 2, 3, 5, 10, 20, and 30 years. CML, chronic myeloid leukemia.

Table 1 shows the baseline characteristics of the 15 cancer cohorts. The sample sizes varied across the cohorts. The largest group was prostate cancer aged 70 to 99 y with *n* = 29,749, and the smallest was CML aged 60 to 69 y, *n* = 218. Melanoma patients aged 18 to 59 y had the highest 10-year and lifetime or 40-y survival outcomes (10-y RMST = 8.70 y, survival proportion at 10 y = 78.79%, 40-y RMST = 27.26 y, and survival proportion at 40 y = 37.95%). CML patients aged 70 to 99 y had the lowest 10-y and lifetime or 40-y survival outcomes (10-y RMST = 1.88 y, survival proportion at 10 y = 1.08%, and LE = 1.90 y). Across all cancer cohorts, the proportions of censoring ranged from 33.06% to 93.64% at 2 y and from 1.08% to 78.89% at 10 y. The K-M survival functions of an observed period of 40 y and numbers of patients at risk at selected time points are presented in Figure 1.

**Table 1:** Baseline characteristics and observed survival outcomes of cancer cohorts, by cancer site and age group, diagnosis in Sweden during 1981-1990 with follow-up until 2020.

				Observed Outcomes							
Cancer Cohort				10 y		Lifetime or 40 y		Censoring Proportion at Time Points (%)			
Type	Age Group (y)	Median Age (y)	n	RMST (y)	Survival (%)	LE (y)	Survival (%)	2 y	3 y	5 y	10 y
Colon	18–59	53	3,627	5.59	44.20	14.38	9.19	64.52	57.29	50.23	44.36
	60–69	65	6,230	5.19	36.51	9.16	0.00	62.02	55.51	46.61	36.57
	70–99	77	14,826	4.05	18.65	5.04	0.00	55.22	46.47	35.38	18.70
Breast	18–59	49	15,991	8.10	67.44	21.63	19.36	91.75	86.88	78.88	67.53
	60–69	65	10,996	7.82	60.44	14.52	0.00	90.31	85.46	76.78	60.49
	70–99	77	16,782	5.75	28.78	7.39	0.00	78.32	69.16	54.30	28.73
Melanoma	18–59	45	5,662	8.70	78.79	27.26	37.95	93.64	90.43	85.39	78.89
	60–69	65	2,638	7.63	60.51	14.67	0.00	87.45	81.73	74.03	60.58
	70–99	76	3,194	5.53	28.66	7.29	0.00	74.20	64.84	50.94	28.71
Prostate	18–59	57	1,808	6.35	38.65	10.59	1.62	82.58	73.34	58.74	38.83
	60–69	66	10,423	6.10	33.74	8.49	0.00	80.74	71.41	57.37	33.84
	70–99	77	29,749	4.47	13.90	5.02	0.00	68.69	56.86	38.94	13.94
CML	18–59	44	359	4.71	20.42	8.16	6.73	69.36	57.66	39.83	20.61
	60–69	66	218	3.21	4.64	3.50	0.00	56.88	43.58	22.48	5.05
	70–99	77	372	1.88	1.08	1.90	0.00	33.06	22.04	10.48	1.08

CML, chronic myeloid leukemia; LE, life expectancy; RMST, restricted mean survival time; y, year(s). If survival proportion was not yet zero by 40 y, the 40-y RMST was reported instead of LE.

### Evaluating 10-y Survival Extrapolation

**Figure 2:**
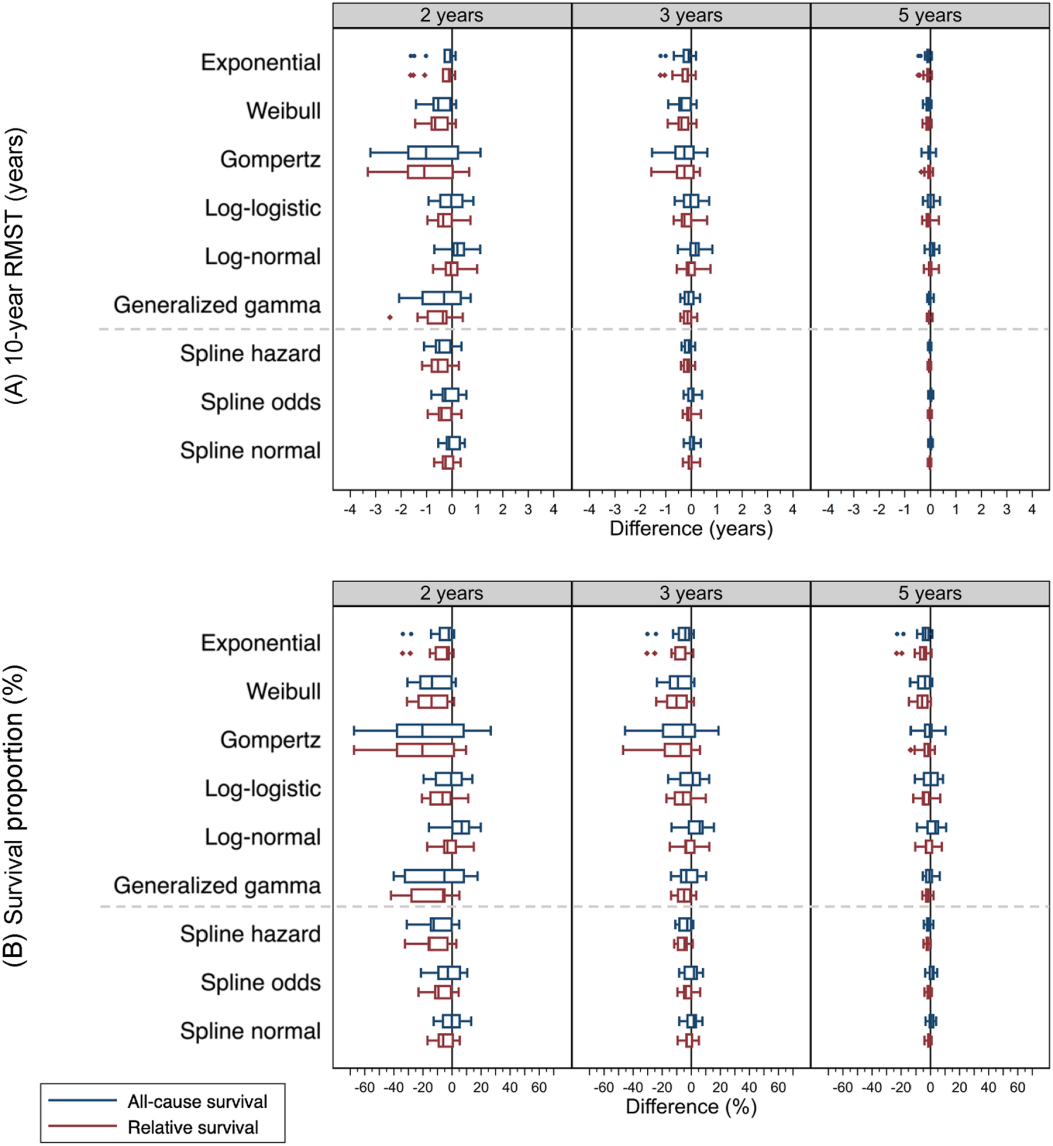
Boxplots show difference (extrapolated minus observed) for (A) 10-year restricted mean survival time (RMST) and (B) survival proportions at 10 years across 15 cancer cohorts, by model, survival framework, and follow-up time used for extrapolation. The extrapolated values were retrieved from models fitted to 2, 3, and 5 years of follow-up data and predicted to 10 years. The observed values were from Kaplan-Meier survival estimates of 10 years. Boxplots in blue represent models using an all-cause survival framework, while those in red are using a relative survival framework.

Figure 2 shows the box plots of difference (extrapolated minus observed) for 10-y RMST (Figure 2A) and survival proportions at 10 y (Figure 2B) across cancer cohorts, by model, and survival framework at which 2-, 3-, and 5-y follow-up time were used for extrapolation to 10 y. For 10-y RMST, the actual differences were provided in dot plots in Supplementary Appendix Figure A1 and presented in numerical values in Supplementary Appendix Table B1; for survival proportions at 10 y, dot plots and numerical values can be found in Supplementary Appendix Figure A2 and Supplementary Appendix Table B2, separately.

Given extrapolation from 2 y, spline models generally had differences of predicted 10-RMST within ±1 y for both an ASF and an RSF, except 4 predicted values by the spline hazard model: −1.10 y in an ASF and −1.14 y in an RSF for breast cancer patients aged 18 to 59 y, and −1.10 y in an ASF and −1.17 in an RSF for melanoma patients aged 60 to 69 y. On the other hand, SPMs had a higher frequency of difference outside ±1 y across 15 cancer cohorts within either framework. With a follow-up of 5 y, all the SPMs and spline models in either survival framework predicted 10-RMST similarly well. The largest difference was −0.49 y for colon aged 18 to 59 y with the exponential model within an RSF (Figure 2A, Supplementary Appendix Figure A1, and Supplementary Appendix Table B1).

With regard to the predicted survival proportions at 10 y, with 2 y of follow-up, all the models in either survival framework generally produced inaccurate extrapolations with the range between −67.42% and 26.68%. With extrapolation from 5 y, the spline models within either framework generated similarly accurate extrapolations with differences ranging from −4.88% to 4.61%. On the contrary, the SPMs within either framework had greater differences (Figure 2B, Supplementary Appendix Figure A2, and Supplementary Appendix Table B2).

### Evaluating Lifetime or 40-y Survival Extrapolation

**Figure 3:**
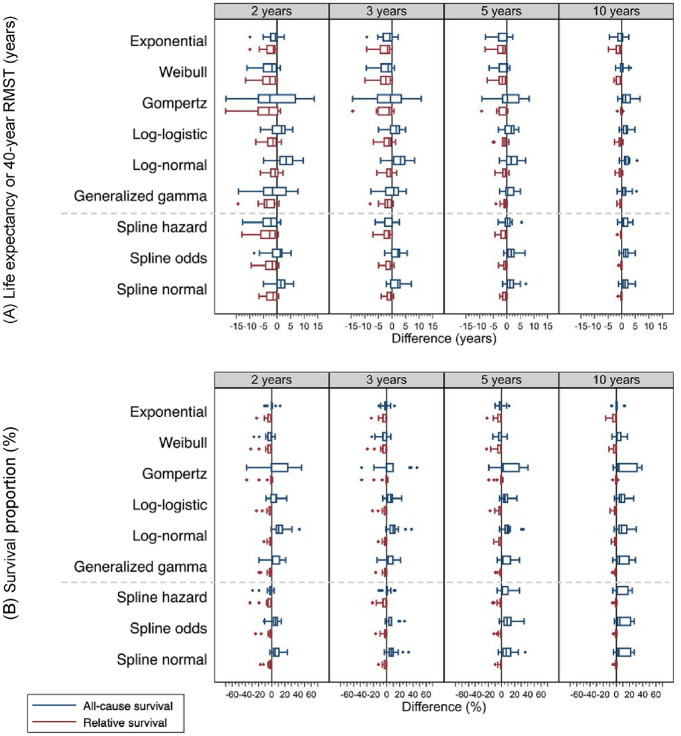
Boxplots show difference (extrapolated minus observed) for (A) life expectancy (LE) or 40-year restricted mean survival time (RMST) (years) and (B) survival proportion (%) at 40 years across 15 cancer cohorts, by model, survival framework, and follow-up time used for extrapolation. The extrapolated values were retrieved from models fitted to 2, 3, 5, and 10 years of follow-up data and predicted to lifetime or 40 years. The observed values were from Kaplan-Meier survival estimates of 40 years. Boxplots in blue represent models using an all-cause survival framework, while those in red are using a relative survival framework. If survival proportion was not yet zero by 40 years, 40-year RMST was evaluated instead of LE.

Similar to the evaluations at 10 y (Figure 2), we present the lifetime difference (or 40 y) in box plots (Figure 3), precision of survival extrapolation on 2 outcomes: LE (or 40-y RMST) (Figure 3A, Supplementary Appendix Figure A3, and Supplementary Appendix Table B), and survival proportions at lifetime (or 40 y) (Figure 3B, Supplementary Appendix Figure A4, and Supplementary Appendix Table B4).

Extrapolations from 2 y are generally imprecise for all the models in either an ASF or an RSF. Given extrapolating from 3 y, most of the spline models within an RSF predicted a difference lower than 5 y, with the largest difference being −7.00 y for breast cancer aged 18 to 59 y extrapolating from the spline model on the hazard scale. However, ASF models and the SPMs within an RSF had wider ranges in difference. Given extrapolating from 5 y, most of the SPMs and the spline models had a difference lower than 5 y, with the largest being −9.28 y for breast cancer aged 18 to 59 y by the Gompertz model within an RSF. With 10 y, under an RSF, extrapolations by both the SPMs and the spline models produced smaller differences, with the largest difference being −4.95 y for colon cancer aged 18 to 59 y extrapolating from the exponential model. Among the spline models, the differences ranged from −1.62 y to 0.05 y. On the other hand, despite using 10 y, extrapolations by all models within an ASF generated a wider range of difference from −4.53 to 6.75 y (Figure 3A, Supplementary Appendix Figure A3, and Supplementary Appendix Table B3).

For survival proportions at lifetime (or 40 y), on the condition that the predicted survival proportion deviated from the observed, extrapolations within an RSF mostly underestimated the survival, while ASF models mostly overestimated the survival, with the highest difference 46.00% for colon cancer aged 60 to 69 y extrapolating from the Gompertz using 3 y. With 10 y, extrapolation within an RSF, especially using the spline models, agreed well with the observed survival, with the largest difference being −16.16% for melanoma aged 18 to 59 y using the exponential model (Figure 3B, Supplementary Appendix Figure A4, and Supplementary Appendix Table B4).

### Evaluating the Extrapolation at Selected Scenarios

Table 2 summarizes the number of cancer cohorts whose absolute differences (absolute value of extrapolated minus observed) were relatively small. This is another approach to represent the precision of survival extrapolation. For 10-y survival outcomes (10-y RMST and survival proportions at 10 y), most parametric models extrapolated in either survival framework did not have distinct differences to predict both 10-y RMST within 0.1 y of difference under the same parametric model and follow-up time. With 2 or 3 y of follow-up time, none of the models within either framework had predictions within 0.1 y for more than half of the 15 cohorts. Given extrapolation from 5 y, among the SPMs, both the Gompertz model and the generalized gamma model within an RSF outperformed the best with predictions on within 0.1 y for 11 of 15 cancer cohorts; among the spline models, the spline normal within an ASF performed the best with predictions within 0.1 y for 15 of 15 cohorts. With regard to survival proportions at 10 y, with 2 or 3 y, all models in either framework had predictions within 1% for less than 5 of 15 cohorts. With 5 y of time, the spline odds and the spline normal within an RSF predicted within 1% survival for 7 and 6 of the 15 cohorts, respectively.

**Table 2:** Number of cancer cohorts whose absolute differences (absolute value of extrapolated minus observed) are relatively small, presented by model, survival framework, and follow-up time used for extrapolation: (a) within 0.1 years from the observed 10-year restricted mean survival time (RMST); (b) 1 % survival proportion at 10 years; (c) 1 year from the observed life expectancy (LE) or 40-year RMST; (d) 1 % at lifetime or 40 years.

	Follow-up Time (y)	Difference at 10 y								Difference at Lifetime or 40 y									
		(a) ≤0.1 y of RMST				(b) ≤1% of Survival Proportion				(c) ≤1 y of LE					(d) ≤1% of Survival Proportion				
		2	3	5	Total	2	3	5	Total	2	3	5	10	Total	2	3	5	10	Total
(Total cohorts)	Survival framework	(15)	(15)	(15)	(45)	(15)	(15)	(15)	(45)	(15)	(15)	(15)	(15)	(60)	(15)	(15)	(15)	(15)	(60)
Standard parametric models																			
Exponential	All-cause	6	6	7	19	4	3	4	11	8	8	8	8	32	8	9	9	10	36
	Relative	6	3	7	16	1	1	2	4	5	5	6	8	24	10	10	10	10	40
Weibull	All-cause	3	3	7	13	2	1	3	6	5	6	8	9	28	8	8	6	7	29
	Relative	2	3	5	10	1	1	2	4	4	5	7	9	25	10	10	10	10	40
Gompertz	All-cause	2	3	8	13	2	3	1	6	2	3	4	6	15	6	6	3	5	20
	Relative	2	3	11	16	0	2	4	6	6	7	9	14	36	10	10	10	12	42
Log-logistic	All-cause	4	5	6	15	3	1	2	6	3	3	2	5	13	0	1	1	0	2
	Relative	3	3	6	12	3	3	3	9	5	5	8	10	28	10	10	10	11	41
Log-normal	All-cause	4	4	8	16	2	1	2	5	3	4	4	5	16	2	2	1	2	7
	Relative	2	2	9	13	0	0	2	2	8	9	9	11	37	10	10	10	11	41
Generalized gamma	All-cause	1	2	10	13	1	2	3	6	0	4	8	5	17	4	4	3	4	15
	Relative	0	4	11	15	0	2	3	5	6	7	9	13	35	10	10	10	11	41
Spline models																			
Spline hazard	All-cause	3	7	14	24	2	2	4	8	3	7	8	8	26	7	7	5	6	25
	Relative	2	4	11	17	0	3	4	7	5	6	8	14	33	10	10	10	11	41
Spline odds	All-cause	0	5	14	19	0	2	3	5	1	3	4	4	12	1	1	2	3	7
	Relative	2	3	14	19	2	1	7	10	6	9	11	14	40	10	10	11	13	44
Spline normal	All-cause	1	7	15	23	1	1	3	5	4	6	5	4	19	1	2	2	3	8
	Relative	2	5	14	21	0	1	6	7	7	8	11	14	40	10	10	11	13	44

LE, life expectancy; RSMT, restricted mean survival time; y, year(s). If survival proportion was not yet zero by 40 y, 40-y RMST was reported instead of LE.

For long-term survival outcomes, models within an RSF in general had higher frequency to predict LE (or 40-y RMST) within 1 y of difference than models within an ASF. In terms of predicting survival proportions at lifetime (or 40 y) of less than 1%, irrespective of the follow-up time employed for extrapolation, all models within an RSF showed frequencies equivalent to or greater than 10 of 15 cohorts. This frequency was notably higher than the same models in an ASF. Among these models, both the spline odds and the spline normal had the highest frequency, 13, to predict within 1% as extrapolating from a 10-y follow-up.

### Visual Evaluation of Extrapolated Survival and Hazard Functions

**Figure 4:**
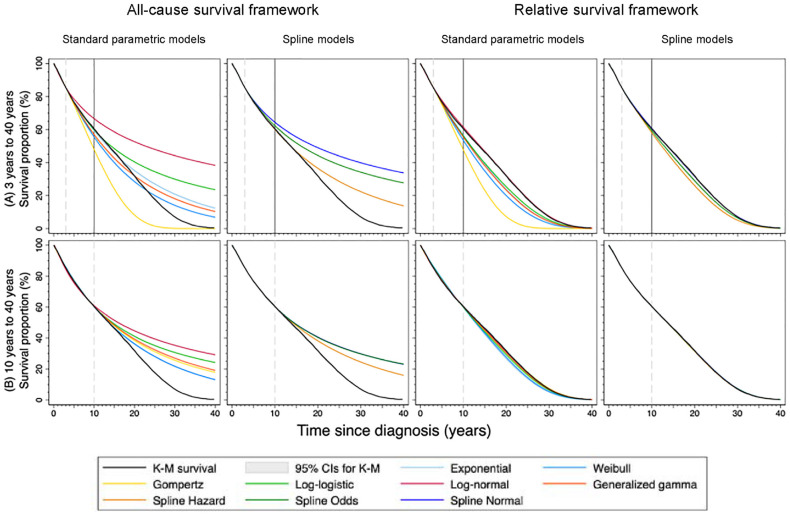
Extrapolated survival curves (A) from 3 years to 40 years and (B) from 10 years to 40 years for breast cancer aged 60-69 years by model, survival framework, follow-up time use for extrapolation. The observed values (black lines) with 95% confidence intervals (shaded areas) were from Kaplan-Meier survival estimates of 40 years. The vertical solid line on plot (A) indicates 10 years. K-M, Kaplan-Meier; CIs, confidence intervals.

We selected breast cancer patients aged 60 to 69 y as an example to present the extrapolated survival functions using either SPMs or spline models within an ASF or an RSF for 10-y survival or lifetime survival in Figure 4. Figure 4A shows the lifetime extrapolated survival functions using 3 y of follow-up. For evaluating 10-y survival outcomes, the results showed that survival extrapolations within an RSF did not necessarily predict more accurate survival than an ASF. For lifetime survival outcomes, we observed that the models within an RSF, in general, agreed better with the observed K-M survival curve compared with the same model within an ASF (Figure 4A). Similar examples can be found in other cancer cohorts (Supplementary Appendices C and D). Despite using a longer follow-up of 10 y (Figure 4B), the extrapolation of all models within an ASF overall overestimated survival. In contrast, most of the models an RSF agreed well with the observed, especially using the spline models. To assist visual assessment for all extrapolations to 10 y, we included the extrapolated survival and hazard functions using an ASF in Supplementary Appendix C and using an RSF in Supplementary Appendix D. For lifetime (or 40-y) survival outcomes, all extrapolated survival and hazard functions within an ASF and an RSF can be found in Supplementary Appendices E and F, respectively.

## Discussion

Survival extrapolation is regularly practiced for predicting survival outcomes in health economic evaluations. An advantageous model should ideally fit the survival during the observed period well and extrapolate unbiased survival beyond follow-up. As a result, it is important to select models that adequately capture the hazard functions and make reasonable predictions.^5^ Flexible parametric spline models were developed to sufficiently capture the hazard functions with potentially complex shapes,^22,26^ but they do not guarantee that the extrapolations are always credible. Despite a longer follow-up period, extrapolating survival using either SPMs or spline models within an ASF may still generate inaccurate predictions on survival estimates at lifetime (Figures 3 and 4). The underlying reason was that it may be difficult to find a suitable parametric distribution within an ASF to describe the hazard function both in the observed and extrapolation period. Among the 15 cancer cohorts, most of the observed all-cause hazard functions were increasing right after time since diagnosis for a short period, decreasing after the spike, and eventually monotonically increasing again. Some exceptions were monotonically increasing functions over time, for example, age groups 70 to 99 y for breast cancer, melanoma, and prostate cancer (Supplementary Appendices E and F).

For multiple types of cancer, the relative survival function tends to drop substantially after cancer diagnosis and later approach a plateau over time. In other words, the excess hazard function spikes initially and approximates constant or even zero in the long run.^27^ This specific characteristic of cancer survival patterns justifies extrapolating spline models within an RSF, because, for spline models, the log cumulative excess hazard beyond the last boundary knot of restricted cubic splines is a linear trend, which makes the excess hazard function in the tail behave like a Weibull distribution.^6^

In this study, we maintained consistency across all the spline models by selecting 4 internal knots, 5 df, for each model. Previous studies concluded that with a sufficient number of knots used in spline models fitted on log cumulative hazard scale, at least greater than 1 internal knot (2 df), the ability of splines to approximate complex hazard functions does not heavily depend on the correct choice of the number of knots.^28,29^ In addition, Andersson et al.^6^ investigated how sensitive the extrapolation is to the chosen number of knots, 5 to 7, that is, 3 to 5 internal knots in the spline models. The results suggested that the extrapolations are not very sensitive to the number of knots.

For 10-y survival outcomes, extrapolations within an RSF were not especially more successful than the same parametric model within an ASF because the extrapolated expected hazard may not yet explain a great part of the all-cause hazard. Instead, it is more important to select models that can capture the hazard shape and predict the shape in the near future, for example, spline models.

For lifetime (or 40-y) survival outcomes, survival extrapolations carried out by models within an RSF generally agreed well with the observed data, especially using spline models. The underlying reason is that the expected hazard from the GPMRs may explain a greater part of the long-term all-cause hazard. In addition, the spline models capture and reasonably predict the underlying excess hazard functions. For predicted survival proportions at lifetime, proportions despite longer follow-up, most of the models within an ASF overestimated survival due to the fact that the long-term extrapolated all-cause hazards were underestimated. On the contrary, the models within an RSF leaned toward underestimating survival, since they may overestimate excess hazards.

### Strengths

A major strength of this study is comparing the extrapolated survival functions with data from a population-based cancer registry of empirical 40-y follow-up. We extended the study by Gray et al.^10^ by investigating extrapolation using both SPMs and spline models within not only an ASF but also an RSF. We evaluated both 10-y survival outcomes and lifetime (or 40-y) outcomes. Gray et al. right-censored the survival data at 20%, 35%, and 50% survival points and extrapolated to 10 y. They found that of the 45 cancer cohorts, the spline models with the lowest Akaike information criterion (AIC) had 23 groups that predicted within 1-mo difference from the observed 10-y RMST, while the SPMs with the lowest AIC had only 9. Instead, our study right-censored the survival data at different time points. Our motivation was that in HTA, clinical trial data are usually followed up until a certain time instead of a certain censoring proportion. For 10-y RMST, within an ASF, we also observed that the spline models generally had a higher frequency of predicting difference within 0.1 y than the SPMs did. We reproduced the spline models within an RSF by Andersson et al.^6^ using different cancer cohorts from the same cancer registry. For predicting survival to lifetime, Andersson et al.^6^ showed that extrapolating the spline models within an RSF to lifetime using 10 y of follow-up had a difference of −0.23 y for colon cancer aged 60 to 69 y, while using an ASF had a difference of 2.13 y. Our study presented that, for colon cancer aged 60 to 69 y, the spline models within an RSF using 10 y had differences ranging from −0.09 to −0.08 y, and an ASF had differences ranging from 3.37 to 3.74 y (Supplementary Table B3).

Sweeting et al.^12^ investigated the use of excess hazard methods in survival extrapolation. They applied SPMs within an excess hazard framework, that is, the RSF in our study. They used breast cancer survival data with a maximum follow-up of 7.3 y. Our study extended this study by including not only SPMs but also spline models within an all-cause hazard (all-cause survival) and an excess hazard (relative survival) framework. Furthermore, considering the heterogeneity of cancer survival, we investigated the extrapolation performance by using a real-word cancer registry data of 5 cancer types across 3 age groups with a maximum follow-up of 40 y.

Another study by van Oostrum et al.^17^ assessed the incorporation GPMRs for extrapolating survival in HTA. They recommended the additive hazards approach, that is, extrapolation within an RSF in our study. In addition, they investigated a variety of other approaches, including converging hazards (also known as imposing statistical cure), external additive hazards, and proportional hazards, which are plausible only under certain scenarios.^17^ Our study corroborated this study by including the generalized gamma model and the spline models that incorporate GPMRs into the investigation. We showed that only certain SPMs, such as the Gompertz model, performed similarly well as the spline models when extrapolating within an RSF to lifetime.

### Limitations

This study, however, has certain limitations. First, the generalizability of this study to health economic evaluations in randomized clinical trial settings may depend on the hazard shape of the disease of interest. For example, the all-cause hazard function may have multiple turning points. The study populations were selected from various cancer sites from the nationwide Swedish Cancer Registry. However, we argue that a total of 15 cancer cohorts had a wide range of heterogeneity in the underlying hazard functions over time. Thus, our results may provide valuable insights into evaluating survival extrapolations on diseases characterized by other complex hazard shapes. Further evaluation is needed when considering the further advancement of treatments on cancer survival. Moreover, additional analyses are require to generalize the findings to other diseases than cancers. Second, clinical trial data usually have much smaller sample sizes than population-based disease registers do. Consequently, the accuracy of extrapolation may need to be addressed under these data-limited scenarios. However, our results also contained chronic myeloid leukemia, which had similarly lower sample sizes (less than 400 in each age group) than other cancer types. Kearns^30^ conducted a simulation study to evaluate the impacts of follow-up time used and sample sizes on extrapolation by different models, including spline models. The author concluded that varying lengths of follow-up had a greater influence on survival extrapolation than varying sample sizes in the defined simulation settings.^30^ We suggest that future research should extend this study to investigate the uncertainty on extrapolated survival estimates due to sample size reduction in both contexts of population-based disease registers and clinical trials. Third, the uncertainty in the extrapolated survival estimates can also come from the expected hazards. In this study, we assumed that the expected hazards, obtained from the GPMRs, were based on the whole general population in Sweden and constant for the same age, sex, and calendar year. However, it has been shown that if estimates of expected measures are not based on the entire population, then the uncertainty in GPMRs should be taken into account.^31^

Other models, such as cure fraction models^32–35^ and mixture-cure models,^9,36^ were also applied to evaluate survival extrapolation in HTA. This article mainly focuses on exploring fitting survival data with a variety of parametric distributions within an ASF and an RSF. Discussion on cure fractions and their impact on survival prediction is beyond scope. Hwang et al.^37^ proposed the rolling extrapolation algorithm to extrapolate the logit transformation of the relative survival ratio. This algorithm also incorporates spline models and the RSF. It has been applied to estimate loss of LE due to cancer^38,39^ as well as other diseases.^40,41^ Further investigations may compare this approach with other existing survival extrapolation methods, especially within an RSF.

Predicting survival from short follow-up time is a demanding task in essence. To select an appropriate model, one should consider not only the goodness-of-fit of the model during observed time but also the credibility of extrapolations beyond follow-up.^42^ Special caution should always be paid as extrapolating survival to lifetime for younger patients or from shorter follow-up time. To enhance the credibility of the extrapolation by the chosen model, researchers should carefully select the model and conduct rigorous sensitivity analysis.

## Conclusions

This study showed the performance of survival extrapolations using SPMs and flexible parametric spline models within an ASF and an RSF for 10-y and lifetime survival outcomes. For survival extrapolation to 10 y, spline models generally outperformed SPMs. However, using an ASF or an RSF showed no distinct difference. For survival extrapolation to lifetime, we recommend relative survival extrapolation for estimating LE, especially using spline models. With limited follow-up, models within an ASF tended to overestimate survival proportions at lifetime; models within an RSF may underestimate instead.

## Supplemental Material

sj-pdf-1-mdm-10.1177_0272989X241227230 – Supplemental material for Comparing Survival Extrapolation within All-Cause and Relative Survival Frameworks by Standard Parametric Models and Flexible Parametric Spline Models Using the Swedish Cancer Registry Supplemental material, sj-pdf-1-mdm-10.1177_0272989X241227230 for Comparing Survival Extrapolation within All-Cause and Relative Survival Frameworks by Standard Parametric Models and Flexible Parametric Spline Models Using the Swedish Cancer Registry by Enoch Yi-Tung Chen, Yuliya Leontyeva, Chia-Ni Lin, Jung-Der Wang, Mark S. Clements and Paul W. Dickman in Medical Decision Making

sj-pdf-2-mdm-10.1177_0272989X241227230 – Supplemental material for Comparing Survival Extrapolation within All-Cause and Relative Survival Frameworks by Standard Parametric Models and Flexible Parametric Spline Models Using the Swedish Cancer Registry Supplemental material, sj-pdf-2-mdm-10.1177_0272989X241227230 for Comparing Survival Extrapolation within All-Cause and Relative Survival Frameworks by Standard Parametric Models and Flexible Parametric Spline Models Using the Swedish Cancer Registry by Enoch Yi-Tung Chen, Yuliya Leontyeva, Chia-Ni Lin, Jung-Der Wang, Mark S. Clements and Paul W. Dickman in Medical Decision Making

sj-pdf-3-mdm-10.1177_0272989X241227230 – Supplemental material for Comparing Survival Extrapolation within All-Cause and Relative Survival Frameworks by Standard Parametric Models and Flexible Parametric Spline Models Using the Swedish Cancer Registry Supplemental material, sj-pdf-3-mdm-10.1177_0272989X241227230 for Comparing Survival Extrapolation within All-Cause and Relative Survival Frameworks by Standard Parametric Models and Flexible Parametric Spline Models Using the Swedish Cancer Registry by Enoch Yi-Tung Chen, Yuliya Leontyeva, Chia-Ni Lin, Jung-Der Wang, Mark S. Clements and Paul W. Dickman in Medical Decision Making

sj-pdf-4-mdm-10.1177_0272989X241227230 – Supplemental material for Comparing Survival Extrapolation within All-Cause and Relative Survival Frameworks by Standard Parametric Models and Flexible Parametric Spline Models Using the Swedish Cancer Registry Supplemental material, sj-pdf-4-mdm-10.1177_0272989X241227230 for Comparing Survival Extrapolation within All-Cause and Relative Survival Frameworks by Standard Parametric Models and Flexible Parametric Spline Models Using the Swedish Cancer Registry by Enoch Yi-Tung Chen, Yuliya Leontyeva, Chia-Ni Lin, Jung-Der Wang, Mark S. Clements and Paul W. Dickman in Medical Decision Making

sj-pdf-5-mdm-10.1177_0272989X241227230 – Supplemental material for Comparing Survival Extrapolation within All-Cause and Relative Survival Frameworks by Standard Parametric Models and Flexible Parametric Spline Models Using the Swedish Cancer Registry Supplemental material, sj-pdf-5-mdm-10.1177_0272989X241227230 for Comparing Survival Extrapolation within All-Cause and Relative Survival Frameworks by Standard Parametric Models and Flexible Parametric Spline Models Using the Swedish Cancer Registry by Enoch Yi-Tung Chen, Yuliya Leontyeva, Chia-Ni Lin, Jung-Der Wang, Mark S. Clements and Paul W. Dickman in Medical Decision Making

sj-pdf-6-mdm-10.1177_0272989X241227230 – Supplemental material for Comparing Survival Extrapolation within All-Cause and Relative Survival Frameworks by Standard Parametric Models and Flexible Parametric Spline Models Using the Swedish Cancer Registry Supplemental material, sj-pdf-6-mdm-10.1177_0272989X241227230 for Comparing Survival Extrapolation within All-Cause and Relative Survival Frameworks by Standard Parametric Models and Flexible Parametric Spline Models Using the Swedish Cancer Registry by Enoch Yi-Tung Chen, Yuliya Leontyeva, Chia-Ni Lin, Jung-Der Wang, Mark S. Clements and Paul W. Dickman in Medical Decision Making
