# Supplementary material for: Comparing Survival Extrapolation within All-Cause and Relative Survival Frameworks by Standard Parametric Models and Flexible Parametric Spline Models Using the Swedish Cancer Registry
Source: Med Decis Making. 2024 Feb 5;44(3):269–82. doi: 10.1177/0272989X241227230 (PMC10988990; doi:10.1177/0272989X241227230)
Supplement: sj-pdf-1-mdm-10.1177_0272989X241227230 – Supplemental material for Comparing Survival Extrapolation within All-Cause and Relative Survival Frameworks by Standard Parametric Models and Flexible Parametric Spline Models Using the Swedish Cancer Registry [file sj-pdf-1-mdm-10.1177_0272989X241227230.pdf]

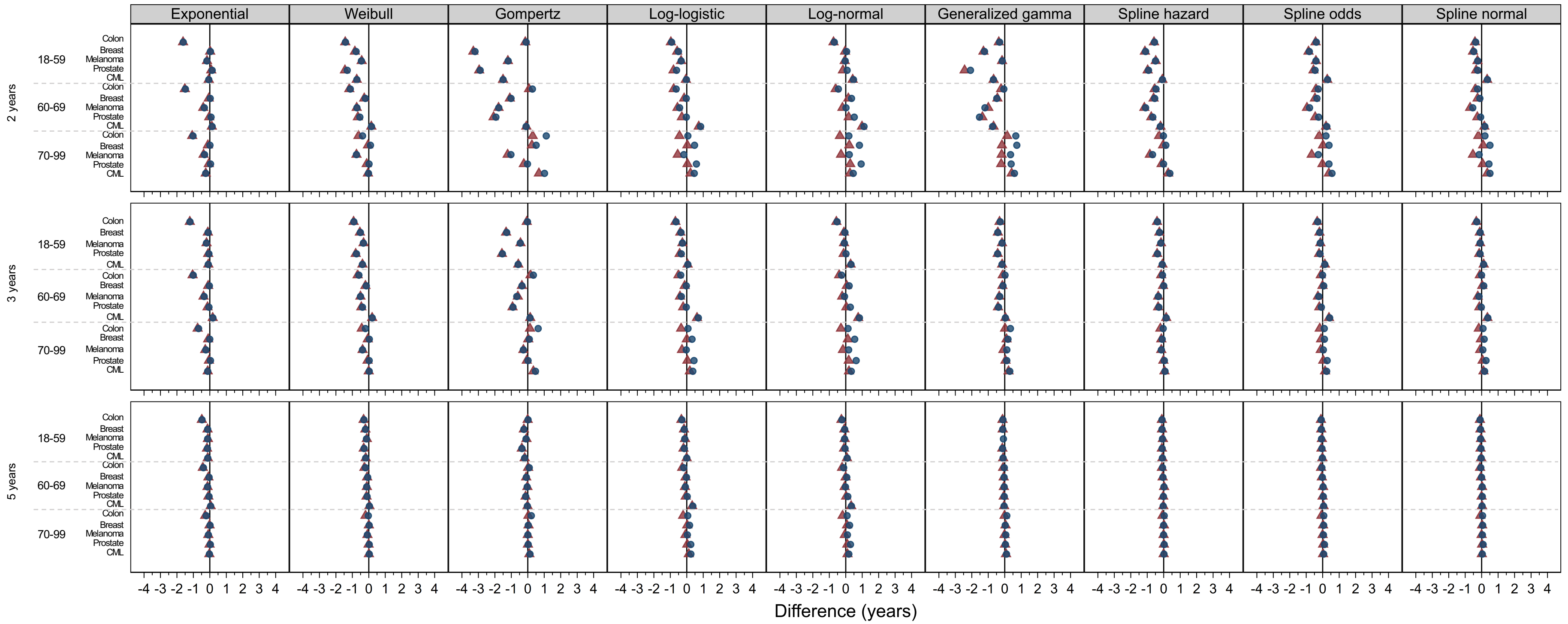

Figure A1. Difference in 10-year restricted mean survival time (RMST) by cancer site, age group, survival framework, model, and follow-up time used for extrapolation. The 10-year observed RMST (years) was the area under the Kaplan-Meier survival curve. The extrapolated values were retrieved from models fitted to each cohort with 2, 3 and 5 years of follow-up data, and extrapolated to 10 years. Difference (years) was calculated as extrapolated minus observed.

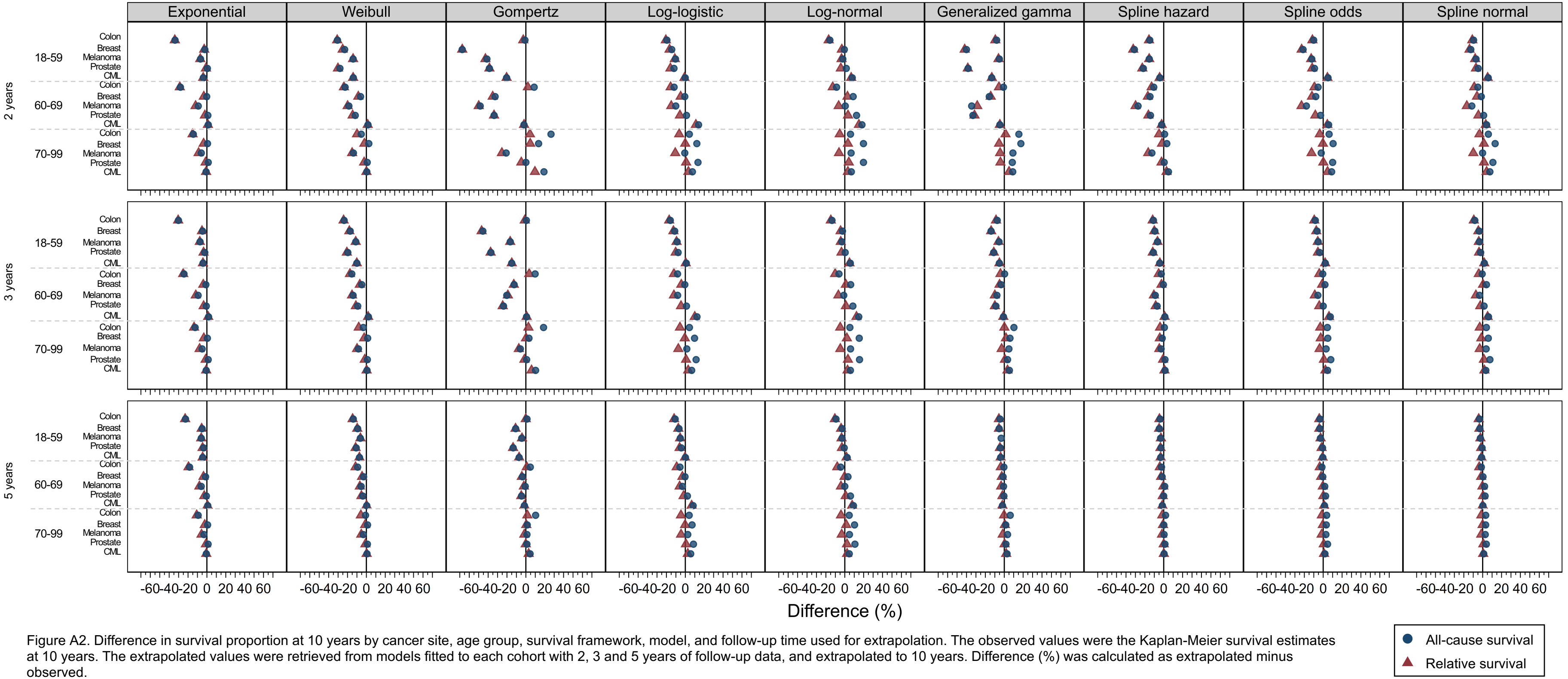

Figure A2. Difference in survival proportion at 10 years by cancer site, age group, survival framework, model, and follow-up time used for extrapolation. The observed values were the Kaplan-Meier survival estimates at 10 years. The extrapolated values were retrieved from models fitted to each cohort with 2, 3 and 5 years of follow-up data, and extrapolated to 10 years. Difference (%) was calculated as extrapolated minus observed.

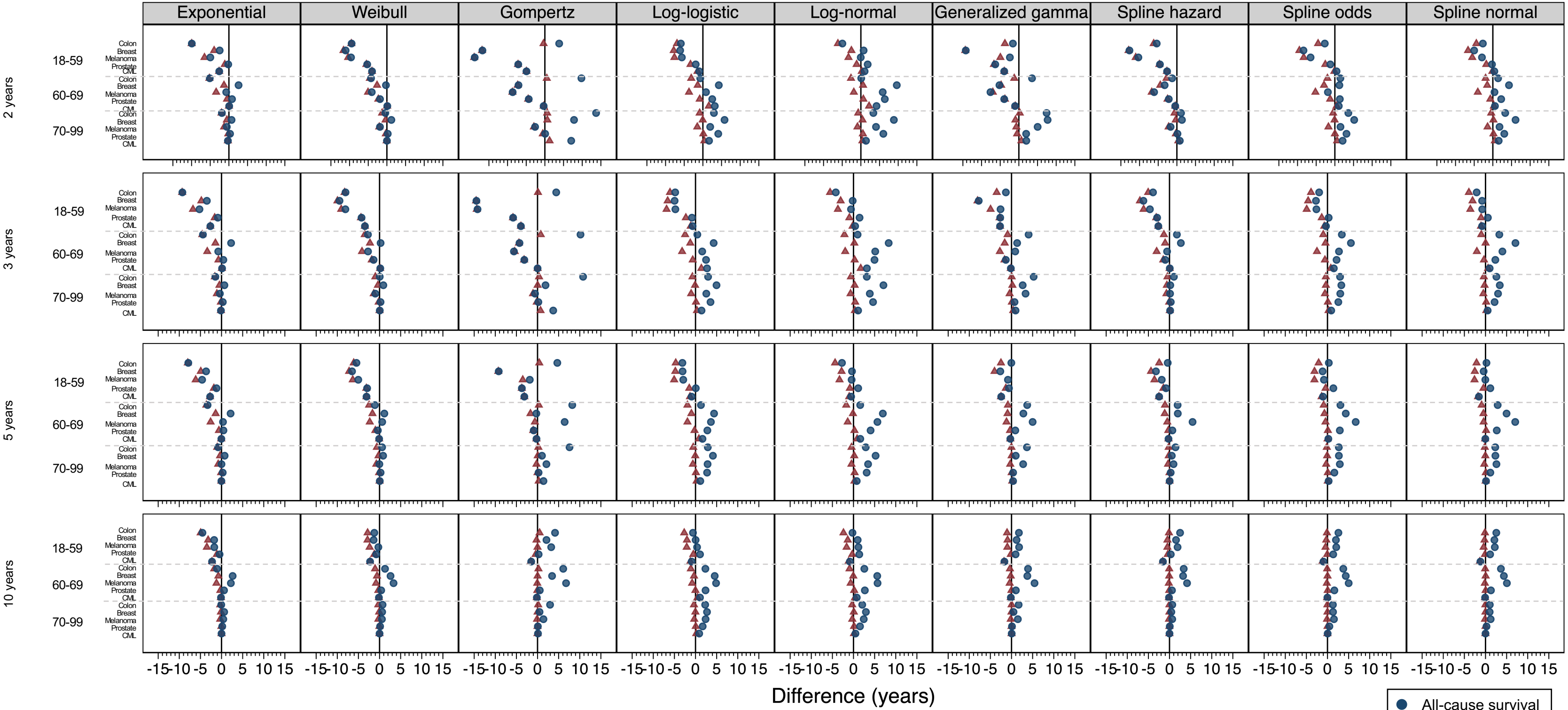

Figure A3. Difference in life expectancy (LE) or 40-year restricted mean survival time (RMST) by cancer site, age group, model, and follow-up time used for extrapolation. The LE or 40-year observed RMST (years) was the area under the Kaplan-Meier survival curve. The extrapolated values were retrieved from models fitted to each cohort with 2, 3, 5, and 10 years of follow-up data, and extrapolated to lifetime or 40 years. Difference (years) was calculated as extrapolated minus observed.

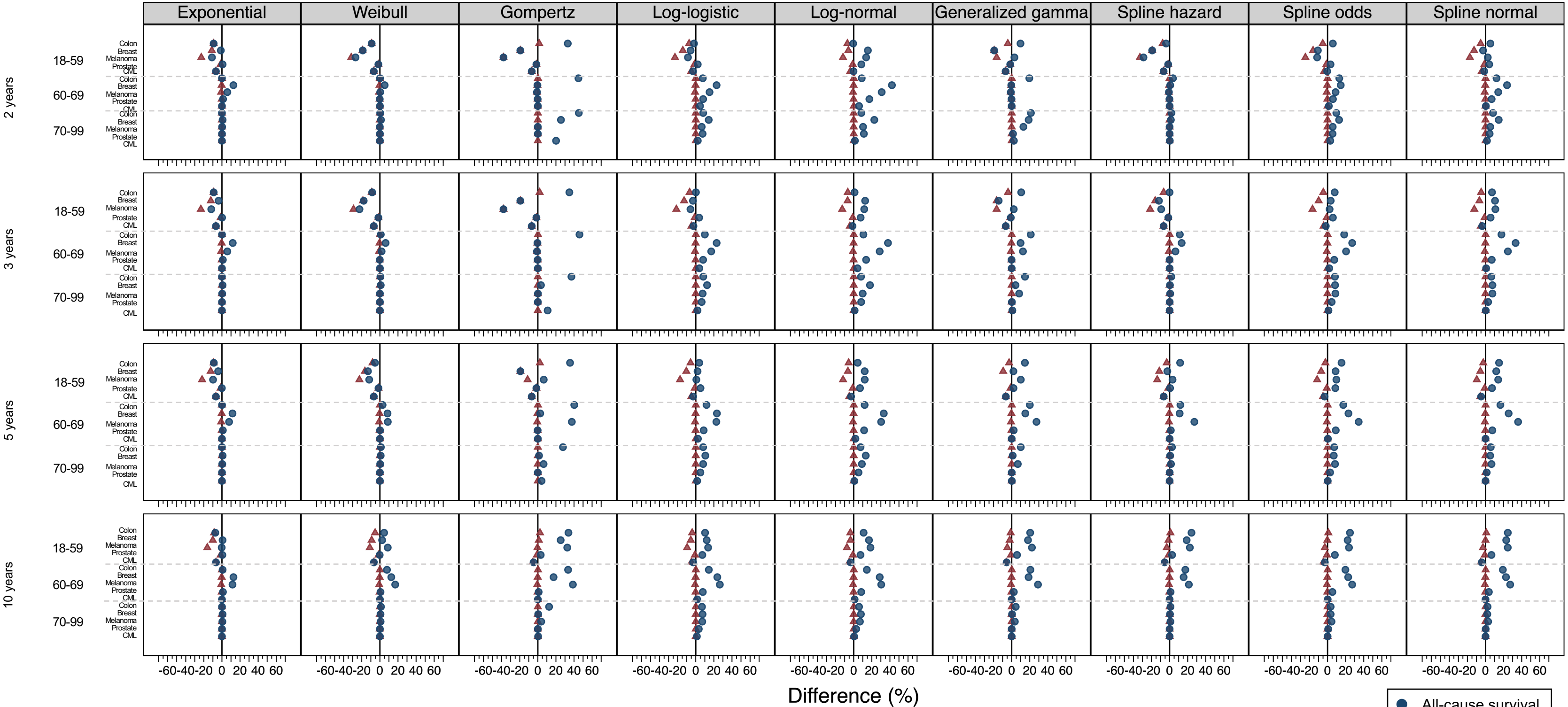

Figure A4. Difference in survival proportion at 40 years by cancer site, age group, model, and follow-up time used for extrapolation. The observed values were the Kaplan-Meier survival estimates at lifetime or 40 years. The extrapolated values were retrieved from models fitted to each cohort with 2, 3, 5, and 10 years of follow-up data, and extrapolated to lifetime or 40 years. Difference (%) was calculated as extrapolated minus observed.
