# Supplementary material for: Comparing Survival Extrapolation within All-Cause and Relative Survival Frameworks by Standard Parametric Models and Flexible Parametric Spline Models Using the Swedish Cancer Registry
Source: Med Decis Making. 2024 Feb 5;44(3):269–82. doi: 10.1177/0272989X241227230 (PMC10988990; doi:10.1177/0272989X241227230)
Supplement: sj-pdf-2-mdm-10.1177_0272989X241227230 – Supplemental material for Comparing Survival Extrapolation within All-Cause and Relative Survival Frameworks by Standard Parametric Models and Flexible Parametric Spline Models Using the Swedish Cancer Registry [file sj-pdf-2-mdm-10.1177_0272989X241227230.pdf]

Table B1. Difference in 10-year restricted mean survival time (RMST) by cancer site, age group, survival framework, model, and follow-up time used for extrapolation. The 10-year observed RMST (years) was the area under the Kaplan-Meier survival curve. The extrapolated values were retrieved from models fitted to each cohort with 2, 3 and 5 years of follow-up data, and extrapolated to 10 years. Difference (years) was calculated as extrapolated minus observed. Bold numbers are values mentioned in the main texts.

	Colon						Breast						Melanoma						Prostate						CML					
Age group	18-59		60-69		70-99		18-59		60-69		70-99		18-59		60-69		70-99		18-59		60-69		70-99		18-59		60-69		70-99	
Survival framework	AS	RS	AS	RS	AS	RS	AS	RS	AS	RS	AS	RS	AS	RS	AS	RS	AS	RS	AS	RS	AS	RS	AS	RS	AS	RS	AS	RS	AS	RS
10-year observed RMST (years)	5.59		5.19		4.05		8.10		7.82		5.75		8.70		7.63		5.53		6.35		6.10		4.47		4.71		3.21		1.88	
Extrapolation from 2 years																														
Exponential	-1.62	-1.63	-1.49	-1.52	-1.02	-1.07	0.04	0.02	0.02	-0.06	0.01	-0.12	-0.18	-0.17	-0.33	-0.40	-0.32	-0.38	0.14	0.08	0.07	-0.04	0.04	-0.06	-0.07	-0.07	0.14	0.12	-0.25	-0.25
Weibull	-1.42	-1.43	-1.13	-1.19	-0.38	-0.63	-0.77	-0.85	-0.21	-0.28	0.10	-0.05	-0.45	-0.43	-0.72	-0.74	-0.75	-0.75	-1.31	-1.45	-0.54	-0.66	0.01	-0.13	-0.72	-0.73	0.16	0.15	-0.02	-0.05
Gompertz	-0.10	-0.15	0.28	0.07	1.12	0.30	-3.21	-3.31	-1.02	-1.09	0.51	0.25	-1.19	-1.21	-1.76	-1.78	-1.02	-1.23	-2.89	-2.95	-1.94	-2.08	-0.02	-0.24	-1.50	-1.50	-0.10	-0.09	1.02	0.67
Log-logistic	-0.92	-0.97	-0.64	-0.80	0.07	-0.44	-0.50	-0.59	-0.03	-0.16	0.47	0.04	-0.34	-0.35	-0.44	-0.57	-0.18	-0.55	-0.62	-0.81	-0.03	-0.30	0.59	0.04	-0.02	-0.06	0.84	0.73	0.45	0.23
Log-normal	-0.70	-0.74	-0.45	-0.62	0.19	-0.35	0.05	-0.05	0.35	0.16	0.83	0.21	-0.03	-0.06	0.01	-0.20	0.21	-0.29	0.09	-0.17	0.52	0.18	0.94	0.27	0.47	0.42	1.11	0.99	0.46	0.26
Generalized gamma	-0.31	-0.36	-0.05	-0.22	0.67	0.15	-1.23	-1.29	-0.48	-0.45	0.73	-0.17	-0.15	-0.16	-1.20	-1.00	0.35	-0.19	-2.08	-2.44	-1.53	-1.36	0.39	-0.22	-0.69	-0.68	-0.74	-0.68	0.59	0.42
Spline hazard	-0.56	-0.59	-0.47	-0.55	-0.02	-0.28	**-1.10**	**-1.14**	-0.55	-0.62	0.13	-0.04	-0.50	-0.49	**-1.10**	**-1.17**	-0.68	-0.84	-0.92	-0.98	-0.66	-0.76	-0.01	-0.13	-0.08	-0.08	-0.21	-0.20	0.37	0.27
Spline odds	-0.39	-0.43	-0.27	-0.41	0.20	-0.21	-0.81	-0.86	-0.35	-0.47	0.38	0.02	-0.41	-0.41	-0.80	-0.96	-0.26	-0.67	-0.46	-0.56	-0.25	-0.46	0.38	-0.02	0.30	0.28	0.25	0.20	0.57	0.37
Spline normal	-0.36	-0.41	-0.25	-0.38	0.20	-0.20	-0.48	-0.53	-0.11	-0.25	0.51	0.09	-0.24	-0.25	-0.54	-0.70	-0.15	-0.53	-0.24	-0.33	-0.06	-0.26	0.44	0.06	0.35	0.33	0.19	0.16	0.50	0.34
Extrapolation from 3 years																														
Exponential	-1.20	-1.22	-1.01	-1.05	-0.68	-0.74	-0.10	-0.12	-0.03	-0.10	0.00	-0.10	-0.21	-0.20	-0.34	-0.39	-0.24	-0.28	-0.06	-0.10	-0.05	-0.14	0.04	-0.05	-0.09	-0.09	0.19	0.17	-0.13	-0.13
Weibull	-0.91	-0.93	-0.60	-0.68	-0.21	-0.43	-0.52	-0.54	-0.15	-0.21	0.03	-0.06	-0.33	-0.32	-0.51	-0.51	-0.38	-0.38	-0.75	-0.80	-0.39	-0.45	0.02	-0.07	-0.39	-0.39	0.21	0.20	0.02	0.00
Gompertz	-0.02	-0.07	0.34	0.16	0.63	0.13	-1.28	-1.32	-0.35	-0.36	0.09	0.03	-0.46	-0.45	-0.67	-0.61	-0.27	-0.27	-1.54	-1.57	-0.90	-0.93	0.02	-0.06	-0.59	-0.58	0.14	0.15	0.47	0.34
Log-logistic	-0.65	-0.69	-0.37	-0.50	0.07	-0.35	-0.35	-0.39	-0.03	-0.13	0.32	0.00	-0.26	-0.27	-0.33	-0.41	-0.03	-0.29	-0.34	-0.43	-0.03	-0.22	0.44	0.04	0.07	0.05	0.70	0.62	0.37	0.19
Log-normal	-0.53	-0.57	-0.25	-0.40	0.14	-0.29	-0.05	-0.11	0.21	0.06	0.54	0.13	-0.08	-0.10	-0.07	-0.22	0.18	-0.17	0.02	-0.13	0.28	0.04	0.63	0.18	0.32	0.29	0.83	0.76	0.34	0.19
Generalized gamma	-0.27	-0.31	0.02	-0.12	0.35	0.02	-0.42	-0.42	-0.11	-0.14	0.20	0.11	-0.16	-0.16	-0.31	-0.33	0.13	-0.08	-0.43	-0.43	-0.38	-0.41	0.12	0.04	-0.17	-0.16	0.04	0.04	0.31	0.23
Spline hazard	-0.38	-0.41	-0.07	-0.15	-0.03	-0.20	-0.25	-0.27	0.00	-0.04	-0.09	-0.15	-0.18	-0.17	-0.32	-0.33	-0.14	-0.15	-0.38	-0.40	-0.29	-0.34	0.04	-0.02	-0.09	-0.08	0.15	0.15	0.08	0.05
Spline odds	-0.30	-0.33	-0.01	-0.11	0.10	-0.18	-0.18	-0.20	0.06	-0.01	0.11	-0.10	-0.15	-0.14	-0.23	-0.29	0.04	-0.12	-0.16	-0.22	-0.08	-0.21	0.27	0.03	0.13	0.13	0.42	0.38	0.24	0.14
Spline normal	-0.30	-0.33	-0.01	-0.11	0.08	-0.18	-0.09	-0.12	0.13	0.03	0.15	-0.05	-0.09	-0.10	-0.16	-0.24	0.07	-0.09	-0.09	-0.15	-0.02	-0.14	0.27	0.06	0.12	0.13	0.37	0.35	0.18	0.11
Extrapolation from 5 years																														
Exponential	-0.48	**-0.49**	-0.39	-0.43	-0.22	-0.28	-0.13	-0.14	-0.05	-0.09	0.01	-0.02	-0.13	-0.12	-0.14	-0.14	-0.09	-0.09	-0.15	-0.17	-0.05	-0.09	0.03	-0.02	-0.13	-0.13	0.05	0.05	-0.03	-0.03
Weibull	-0.30	-0.32	-0.20	-0.27	-0.03	-0.20	-0.20	-0.21	-0.07	-0.10	0.02	0.00	-0.14	-0.12	-0.13	-0.12	-0.09	-0.09	-0.29	-0.31	-0.11	-0.14	0.02	-0.02	-0.19	-0.19	0.04	0.04	0.03	0.00
Gompertz	0.02	-0.01	0.09	0.02	0.21	0.04	-0.24	-0.24	-0.09	-0.10	0.03	0.04	-0.11	-0.08	-0.04	-0.02	0.01	0.00	-0.35	-0.37	-0.13	-0.13	0.03	0.01	-0.19	-0.19	-0.02	-0.02	0.13	0.09
Log-logistic	-0.30	-0.33	-0.18	-0.28	0.06	-0.22	-0.15	-0.17	-0.02	-0.07	0.17	0.03	-0.12	-0.11	-0.09	-0.11	0.04	-0.08	-0.15	-0.20	0.03	-0.07	0.24	0.03	0.00	-0.01	0.37	0.33	0.25	0.12
Log-normal	-0.23	-0.26	-0.13	-0.22	0.08	-0.19	-0.07	-0.09	0.06	-0.01	0.24	0.08	-0.07	-0.07	-0.02	-0.07	0.10	-0.06	-0.04	-0.10	0.13	0.02	0.29	0.09	0.08	0.07	0.34	0.33	0.19	0.11
Generalized gamma	-0.11	-0.14	-0.03	-0.10	0.12	-0.01	-0.12	-0.13	-0.04	-0.06	0.03	0.05	-0.08	-0.05	-0.03	-0.05	0.06	-0.01	-0.13	-0.15	-0.03	-0.06	0.04	0.02	-0.10	-0.10	-0.04	-0.04	0.11	0.08
Spline hazard	-0.10	-0.11	-0.07	-0.11	0.03	-0.08	-0.10	-0.10	-0.05	-0.06	-0.01	0.01	-0.08	-0.05	0.01	0.02	-0.01	-0.01	-0.09	-0.11	-0.02	-0.03	0.03	0.01	-0.08	-0.08	-0.03	-0.02	0.01	0.00
Spline odds	-0.09	-0.11	-0.05	-0.10	0.07	-0.08	-0.08	-0.09	-0.02	-0.05	0.06	0.02	-0.07	-0.04	0.03	0.03	0.05	-0.01	-0.05	-0.07	0.04	-0.01	0.11	0.03	-0.02	-0.02	0.06	0.05	0.06	0.02
Spline normal	-0.09	-0.11	-0.05	-0.10	0.06	-0.08	-0.07	-0.08	-0.01	-0.04	0.06	0.03	-0.06	-0.12	0.03	0.03	0.05	0.00	-0.04	-0.07	0.04	0.00	0.09	0.03	-0.03	-0.03	0.02	0.02	0.04	0.01

AS, all-cause survival; RS, relative survival; RSMT, restricted mean survival time; CML, chronic myeloid leukemia.

Table B2. Difference in survival proportion at 10 years by cancer site, age group, survival framework, model, and follow-up time used for extrapolation. The observed values were the Kaplan-Meier survival estimates at 10 years. The extrapolated values were retrieved from models fitted to each cohort with 2, 3 and 5 years of follow-up data, and extrapolated to 10 years. Difference (%) was calculated as extrapolated minus observed. Bold numbers are values mentioned in the main texts.

	Colon						Breast						Melanoma						Prostate						CML					
Age group	18-59		60-69		70-99		18-59		60-69		70-99		18-59		60-69		70-99		18-59		60-69		70-99		18-59		60-69		70-99	
Survival framework	AS	RS	AS	RS	AS	RS	AS	RS	AS	RS	AS	RS	AS	RS	AS	RS	AS	RS	AS	RS	AS	RS	AS	RS	AS	RS	AS	RS	AS	RS
Survival proportion at 10 years (%)	44.32		36.51		18.65		67.42		60.42		28.70		78.79		60.51		28.66		38.65		33.74		13.90		20.42		4.64		1.08	
Extrapolation from 2 years																														
Exponential	-33.80	-34.00	-28.11	-28.72	-14.45	-15.22	-2.21	-2.87	-0.24	-3.27	0.62	-3.48	-6.76	-7.06	-9.20	-12.00	-5.98	-8.81	0.49	-1.03	1.02	-2.17	1.41	-1.45	-3.80	-3.93	1.44	1.04	-0.86	-0.89
Weibull	-30.66	-30.96	-22.55	-23.82	-5.44	-9.92	-23.03	-25.13	-6.16	-8.55	2.59	-2.15	-14.04	-14.08	-18.55	-19.90	-13.78	-15.10	-28.02	-30.11	-11.81	-14.59	0.89	-2.41	-13.86	-14.06	1.71	1.39	0.45	0.03
Gompertz	-0.99	-2.75	8.77	2.13	**26.68**	4.30	**-67.42**	-67.42	-32.59	-35.13	13.52	4.60	-41.32	-42.88	-48.41	-49.97	-20.89	-25.42	-38.65	-38.65	-33.62	-33.73	0.04	-4.83	-20.41	-20.41	-1.99	-1.89	19.19	9.62
Log-logistic	-19.54	-20.65	-11.89	-15.61	4.22	-6.52	-14.43	-16.71	-0.59	-4.93	12.22	-0.14	-10.45	-11.12	-10.17	-14.92	-0.56	-10.69	-12.06	-15.84	1.23	-5.75	13.42	0.81	-0.13	-0.91	13.98	11.17	7.46	3.26
Log-normal	-15.85	-17.09	-8.73	-12.82	6.01	-5.37	-0.52	-3.16	8.87	2.81	19.80	3.31	-2.13	-3.45	0.48	-6.50	6.68	-6.15	1.56	-4.21	12.46	3.43	19.78	4.23	7.89	6.74	18.17	14.97	6.93	3.28
Generalized gamma	-8.09	-9.50	-0.85	-5.60	15.46	1.34	-40.13	-41.97	-15.84	-14.34	17.61	-5.48	-5.23	-6.10	-34.54	-28.65	9.16	-4.38	-38.33	-38.65	-33.23	-31.57	8.53	-4.04	-13.30	-13.18	-4.64	-4.64	8.96	5.08
Spline hazard	-14.80	-15.62	-10.54	-12.84	0.91	-4.97	-31.10	-32.34	-14.53	-16.82	3.25	-1.80	-15.32	-15.44	-27.21	-29.84	-12.55	-16.44	-21.64	-22.69	-14.06	-16.24	0.48	-2.34	-4.11	-4.33	-2.21	-2.22	4.97	2.97
Spline odds	-10.14	-11.45	-5.43	-9.31	6.27	-3.66	-21.35	-22.86	-7.90	-11.93	10.49	-0.36	-12.12	-12.76	-17.85	-23.01	-2.10	-12.34	-9.26	-11.67	-2.83	-8.37	9.99	0.12	5.07	4.43	5.71	4.35	8.92	4.59
Spline normal	-9.69	-10.97	-5.16	-8.95	6.03	-3.48	-12.60	-13.97	-1.48	-6.02	13.21	1.24	-7.03	-7.99	-11.54	-16.84	-0.12	-9.91	-4.75	-7.11	1.02	-4.51	10.84	1.37	5.87	5.29	4.16	3.29	7.62	4.15
Extrapolation from 3 years																														
Exponential	-30.21	-30.47	-24.28	-25.13	-12.52	-13.62	-4.61	-5.22	-1.11	-3.83	0.49	-3.28	-7.27	-7.56	-9.36	-11.77	-5.06	-7.74	-2.29	-3.57	-0.70	-3.47	1.34	-1.36	-4.09	-4.19	1.72	1.34	-0.74	-0.79
Weibull	-23.64	-24.14	-15.47	-17.40	-3.31	-8.11	-17.14	-18.07	-4.90	-6.96	1.38	-2.36	-11.22	-11.34	-14.09	-15.24	-8.42	-9.88	-19.67	-20.71	-9.17	-11.14	1.00	-1.78	-10.10	-10.18	2.11	1.82	0.67	0.21
Gompertz	0.54	-1.16	9.81	3.53	18.73	2.72	-45.58	-46.90	-12.44	-12.77	3.31	0.17	-16.53	-16.60	-20.21	-19.03	-5.82	-7.42	-37.07	-37.35	-23.68	-24.53	0.83	-1.58	-14.97	-14.93	0.74	0.81	10.32	6.00
Log-logistic	-15.95	-17.10	-8.25	-12.11	4.31	-5.70	-11.28	-12.64	-0.55	-4.27	9.77	-0.60	-8.73	-9.32	-8.14	-12.08	1.55	-7.42	-7.66	-10.24	1.15	-4.57	11.44	0.78	1.01	0.42	12.32	9.99	6.70	3.03
Log-normal	-13.58	-14.80	-6.16	-10.25	5.43	-4.85	-2.47	-4.48	6.22	1.12	15.30	2.21	-3.17	-4.28	-1.12	-6.81	6.11	-4.73	0.41	-3.54	8.57	1.20	15.59	3.29	5.82	4.97	14.79	12.40	5.92	2.93
Generalized gamma	-7.47	-8.75	0.37	-4.06	10.21	0.11	-13.95	-13.90	-3.42	-4.97	6.07	1.81	-5.53	-6.13	-7.81	-9.99	4.91	-2.80	-11.19	-11.09	-8.88	-9.93	3.46	0.72	-5.19	-5.11	-0.86	-1.01	5.40	3.41
Spline hazard	-10.96	-11.75	-2.66	-5.39	0.65	-3.89	-9.45	-10.00	-0.43	-2.40	-1.60	-4.17	-6.54	-6.63	-8.98	-10.44	-2.77	-4.70	-11.00	-11.66	-6.96	-8.51	1.35	-0.70	-4.24	-4.08	1.17	0.97	1.36	0.75
Spline odds	-8.37	-9.50	-0.52	-4.19	4.41	-3.18	-6.51	-7.37	1.85	-1.19	4.85	-2.64	-5.37	-5.76	-5.63	-8.91	3.08	-3.86	-3.81	-5.62	0.05	-4.18	8.05	0.77	2.22	2.01	7.52	6.16	4.58	2.33
Spline normal	-8.30	-9.40	-0.54	-4.12	3.91	-3.13	-3.63	-4.58	4.00	0.40	5.94	-1.41	-3.45	-4.10	-3.36	-7.19	3.66	-3.18	-2.13	-3.83	1.44	-2.57	7.65	1.30	1.75	1.63	6.16	5.24	3.33	1.82
Extrapolation from 5 years																														
Exponential	-22.72	-23.08	-18.36	-19.49	-9.30	-10.85	-5.08	-5.57	-1.42	-3.61	0.72	-2.32	-5.98	-6.06	-6.05	-7.97	-3.21	-5.71	-3.54	-4.56	-0.59	-2.83	1.26	-1.06	-4.41	-4.52	0.89	0.62	-0.61	-0.69
Weibull	-13.90	-14.61	-9.29	-11.54	-0.93	-5.83	-9.31	-9.70	-2.95	-4.56	1.20	-1.34	-6.43	-6.33	-5.57	-6.92	-3.34	-5.41	-10.80	-11.41	-3.83	-5.26	1.07	-1.01	-7.30	-7.39	0.36	0.21	0.69	0.26
Gompertz	1.20	-0.08	4.71	0.99	10.34	1.60	-10.81	-10.94	-3.63	-4.23	1.45	0.42	-4.39	-4.07	-0.63	-2.07	1.15	-1.60	-13.45	-13.55	-4.57	-4.86	1.11	-0.10	-7.09	-7.16	-1.58	-1.56	4.47	2.98
Log-logistic	-10.79	-11.90	-5.66	-9.17	4.10	-4.61	-6.69	-7.41	-0.34	-3.09	7.15	-0.26	-5.36	-5.55	-3.09	-6.01	2.58	-4.51	-4.30	-6.06	2.20	-1.90	8.60	0.69	0.11	-0.34	8.27	6.69	5.67	2.65
Log-normal	-9.37	-10.52	-4.40	-8.01	4.69	-4.01	-2.80	-3.94	3.37	-0.24	10.33	1.54	-2.91	-3.45	0.01	-4.06	4.90	-3.28	-0.60	-3.03	5.98	0.95	10.74	2.33	2.50	1.83	9.13	7.76	4.71	2.45
Generalized gamma	-4.53	-5.65	-0.41	-3.65	6.33	-0.20	-5.35	-5.64	-1.25	-2.67	1.59	0.62	-3.29	-3.09	-0.79	-3.34	3.49	-1.66	-4.18	-4.58	-0.43	-1.99	1.77	0.33	-3.81	-3.87	-1.91	-2.01	2.94	2.03
Spline hazard	-3.93	-4.53	-2.37	-4.13	1.92	-1.55	-4.64	**-4.88**	-1.72	-2.81	0.10	-0.82	-3.31	-3.00	1.03	-0.48	0.35	-1.82	-3.00	-3.48	-0.08	-1.08	1.28	-0.01	-3.19	-3.33	-1.46	-1.53	0.36	0.09
Spline odds	-3.40	-4.15	-1.34	-3.65	3.62	-1.42	-3.67	-4.08	-0.44	-2.19	3.24	-0.37	-2.94	-2.78	1.64	-0.40	2.86	-1.65	-0.68	-1.73	2.51	0.14	**4.61**	0.63	-0.43	-0.70	1.38	0.91	1.82	0.86
Spline normal	-3.43	-4.16	-1.43	-3.66	3.22	-1.42	-2.99	-3.44	0.33	-1.60	3.16	-0.08	-2.44	-2.40	1.97	-0.32	2.73	-1.52	-0.63	-1.60	2.55	0.38	3.83	0.70	-1.14	-1.36	0.24	-0.01	1.13	0.54

AS, all-cause survival; RS, relative survival; RSMT, restricted mean survival time; CML, chronic myeloid leukemia.

Table B3. Difference in life expectancy (LE) or 40-year restricted mean survival time (RMST) by cancer site, age group, model, and follow-up time used for extrapolation. The LE or 40-year observed RMST (years) was the area under the Kaplan-Meier survival curve. The extrapolated values were retrieved from models fitted to each cohort with 2, 3, 5, and 10 years of follow-up data, and extrapolated to lifetime or 40 years. Difference (years) was calculated as extrapolated minus observed. Bold numbers are values mentioned in the main texts.

	Colon						Breast						Melanoma						Prostate						CML					
Age group	18-59		60-69		70-99		18-59		60-69		70-99		18-59		60-69		70-99		18-59		60-69		70-99		18-59		60-69		70-99	
Survival framework	AS	RS	AS	RS	AS	RS	AS	RS	AS	RS	AS	RS	AS	RS	AS	RS	AS	RS	AS	RS	AS	RS	AS	RS	AS	RS	AS	RS	AS	RS
LE or 40-year observed RMST (years)	14.38		9.16		5.04		21.63		14.52		7.39		27.26		14.67		7.29		10.59		8.49		5.02		8.16		3.50		1.90	
Extrapolation from 2 years																														
Exponential	-9.93	-9.97	-5.12	-5.22	-1.88	-1.97	-2.47	-3.95	2.59	-1.32	0.70	-0.53	-4.98	-6.54	-0.72	-3.42	-0.57	-1.27	-0.18	-1.09	0.83	-0.50	0.31	-0.17	-2.59	-2.63	0.07	0.02	-0.27	-0.27
Weibull	-9.45	-9.52	-4.25	-4.55	-0.42	-1.29	-11.06	-11.63	-0.26	-2.64	1.19	-0.35	-9.61	-10.38	-4.05	-5.06	-1.86	-2.05	-5.28	-5.50	-1.84	-2.30	0.22	-0.28	-3.99	-4.01	0.11	0.06	0.00	-0.05
Gompertz	3.83	-0.36	9.78	0.51	13.72	0.61	-16.73	-16.84	-7.07	-7.28	7.81	0.66	-18.81	-18.95	-8.64	-8.69	-2.64	-2.95	-7.14	-7.20	-4.33	-4.47	0.06	-0.55	-4.94	-4.95	-0.34	-0.32	7.05	1.26
Log-logistic	-5.91	-6.93	-0.71	-3.12	2.95	-0.86	-6.09	-7.81	4.27	-1.39	5.80	-0.05	-5.64	-7.62	0.84	-3.73	1.96	-1.50	-1.93	-3.32	2.51	-0.85	4.12	0.11	-0.99	-1.44	3.19	1.59	1.65	0.39
Log-normal	-4.96	-6.16	0.10	-2.66	3.34	-0.71	0.73	-2.54	9.62	0.58	8.84	0.43	-0.03	-3.33	5.85	-1.90	4.05	-0.92	1.82	-1.14	6.41	0.66	6.02	0.54	1.00	0.24	4.18	2.20	1.37	0.41
Generalized gamma	-1.60	-3.74	3.53	-1.24	7.43	0.24	-14.23	-14.37	-5.21	-4.76	7.70	-0.92	-2.41	-4.96	-7.61	-6.97	5.02	-0.68	-6.32	-6.68	-3.92	-3.74	1.95	-0.47	-3.92	-3.91	-1.02	-0.97	1.98	0.68
Spline hazard	-5.41	-6.21	-1.26	-2.77	1.11	-0.64	-12.70	-12.93	-3.27	-4.44	1.36	-0.31	-10.32	-11.03	-6.11	-6.73	-1.69	-2.21	-4.54	-4.68	-2.17	-2.50	0.16	-0.28	-2.62	-2.67	-0.44	-0.43	0.79	0.40
Spline odds	-2.70	-4.49	1.48	-1.99	3.62	-0.46	-8.41	-9.51	1.21	-2.90	5.17	-0.08	-6.51	-8.35	-1.87	-5.21	1.51	-1.72	-1.22	-2.56	1.33	-1.26	3.11	0.01	0.45	-0.15	1.05	0.46	2.07	0.60
Spline normal	-2.67	-4.42	1.40	-1.94	3.32	-0.44	-5.01	-6.65	4.31	-1.50	6.08	0.14	-2.92	-5.53	0.43	-4.04	1.79	-1.42	-0.24	-1.76	2.23	-0.69	3.06	0.16	0.40	-0.13	0.56	0.28	1.56	0.54
Extrapolation from 3 years																														
Exponential	-9.27	-9.33	-4.40	-4.56	-1.45	-1.59	-3.47	-4.79	2.26	-1.44	0.68	-0.50	-5.23	-6.75	-0.78	-3.38	-0.39	-1.11	-0.88	-1.62	0.43	-0.72	0.30	-0.15	-2.64	-2.68	0.13	0.08	-0.14	-0.15
Weibull	-8.06	-8.28	-2.75	-3.50	0.05	-0.99	-9.48	-9.97	0.26	-2.27	0.90	-0.38	-8.09	-9.07	-2.73	-4.15	-1.00	-1.37	-4.26	-4.40	-1.41	-1.84	0.24	-0.20	-3.46	-3.47	0.18	0.14	0.06	0.00
Gompertz	4.43	0.11	10.17	0.77	10.79	0.36	-14.46	-14.54	-4.27	-4.40	1.89	0.02	-14.21	-14.39	-5.59	-5.41	-0.59	-1.06	-5.78	-5.81	-3.13	-3.18	0.20	-0.18	-3.94	-3.93	0.01	0.02	3.71	0.71
Log-logistic	-4.81	-6.05	0.43	-2.46	2.98	-0.72	-4.96	-6.59	4.30	-1.25	4.98	-0.11	-4.77	-6.85	1.58	-3.17	2.56	-1.04	-0.84	-2.29	2.49	-0.66	3.54	0.10	-0.69	-1.13	2.72	1.38	1.42	0.34
Log-normal	-4.21	-5.55	0.96	-2.16	3.15	-0.62	-0.20	-3.05	8.33	0.19	7.08	0.26	-0.60	-3.70	5.14	-1.97	3.85	-0.71	1.43	-0.99	4.97	0.26	4.63	0.40	0.38	-0.23	3.17	1.71	1.09	0.33
Generalized gamma	-1.33	-3.50	4.05	-0.93	5.24	0.04	-7.76	-8.05	1.33	-1.62	2.64	0.21	-2.60	-4.96	0.88	-2.76	3.32	-0.46	-2.67	-2.81	-1.35	-1.66	0.72	0.09	-2.71	-2.72	-0.16	-0.16	0.95	0.40
Spline hazard	-3.91	-5.05	1.74	-1.33	1.04	-0.49	-6.18	**-7.00**	2.69	-1.03	0.18	-0.63	-4.63	-6.13	-0.57	-3.05	0.13	-0.72	-2.94	-3.15	-1.00	-1.47	0.30	-0.08	-2.64	-2.63	0.06	0.04	0.15	0.07
Spline odds	-1.98	-3.90	3.39	-1.01	2.98	-0.39	-2.73	-4.58	5.55	-0.48	3.28	-0.38	-2.63	-4.93	2.71	-2.49	3.03	-0.60	0.28	-1.36	2.15	-0.60	2.56	0.10	-0.36	-0.76	1.49	0.78	0.88	0.26
Spline normal	-2.09	-3.93	3.27	-1.00	2.58	-0.39	-0.81	-3.06	7.09	0.02	3.38	-0.22	-0.77	-3.59	4.00	-2.04	2.97	-0.51	0.56	-1.05	2.33	-0.36	2.15	0.15	-0.80	-1.07	0.99	0.61	0.51	0.19
Extrapolation from 5 years																														
Exponential	-7.86	-7.99	-3.31	-3.59	-0.82	-1.02	-3.67	-4.91	2.14	-1.39	0.73	-0.35	-4.60	-6.12	0.34	-2.60	-0.01	-0.79	-1.18	-1.82	0.45	-0.62	0.28	-0.10	-2.70	-2.73	-0.04	-0.07	-0.04	-0.05
Weibull	-5.47	-6.11	-1.10	-2.47	0.61	-0.63	-6.52	-7.20	1.14	-1.68	0.85	-0.23	-5.04	-6.34	0.63	-2.32	-0.04	-0.76	-2.94	-3.12	-0.39	-0.98	0.25	-0.10	-3.08	-3.09	-0.09	-0.10	0.06	0.02
Gompertz	4.68	0.41	8.23	0.32	7.58	0.20	-9.19	**-9.28**	-0.28	-1.67	1.04	0.06	-1.84	-3.50	6.43	-0.62	2.11	-0.25	-3.70	-3.72	-0.91	-1.03	0.25	0.00	-3.13	-3.14	-0.25	-0.24	1.41	0.29
Log-logistic	-3.11	-4.69	1.28	-1.91	2.92	-0.54	-3.06	-4.79	4.38	-0.98	4.12	-0.06	-2.90	-5.06	3.65	-1.91	2.86	-0.63	0.07	-1.48	2.80	-0.24	2.74	0.09	-0.92	-1.31	1.65	0.79	1.13	0.26
Log-normal	-2.73	-4.37	1.58	-1.72	2.90	-0.47	-0.36	-2.83	6.94	-0.13	5.23	0.16	-0.45	-3.31	5.66	-1.38	3.45	-0.50	1.10	-0.87	4.05	0.22	3.11	0.25	-0.57	-1.01	1.60	0.85	0.76	0.22
Generalized gamma	-0.04	-2.50	3.71	-0.85	3.65	-0.01	-2.65	-4.03	2.81	-0.94	1.00	0.04	-0.85	-3.07	5.00	-1.18	2.76	-0.30	-0.61	-1.34	0.90	-0.35	0.38	0.04	-2.44	-2.49	-0.27	-0.27	0.37	0.17
Spline hazard	-0.42	-2.44	1.92	-1.06	1.42	-0.19	-3.21	-4.39	1.96	-1.13	0.54	-0.17	-1.84	-3.57	5.46	-0.53	0.95	-0.34	-0.83	-1.43	0.68	-0.28	0.28	0.00	-2.45	-2.50	-0.24	-0.24	0.03	0.00
Spline odds	0.31	-2.10	3.06	-0.90	2.68	-0.17	-1.16	-3.08	4.32	-0.73	2.69	-0.08	-0.84	-3.10	6.68	-0.48	2.92	-0.31	1.38	-0.45	2.92	0.10	1.63	0.08	-1.07	-1.40	0.20	0.03	0.27	0.07
Spline normal	0.23	-2.12	2.89	-0.91	2.32	-0.17	-0.44	-2.53	4.99	-0.49	2.35	-0.04	-0.04	-2.60	7.04	-0.44	2.59	-0.29	1.15	-0.48	2.66	0.13	1.15	0.08	-1.56	-1.77	-0.04	-0.09	0.12	0.04
Extrapolation from 10 years																														
Exponential	**-4.53**	**-4.95**	-1.06	-1.73	-0.06	-0.30	-1.75	-3.17	2.63	-0.75	0.63	-0.14	-1.72	-3.47	2.21	-1.20	0.42	-0.32	-0.47	-1.10	0.59	-0.29	0.17	-0.03	-2.24	-2.27	-0.13	-0.14	0.00	-0.01
Weibull	-1.23	-2.82	1.30	-1.05	0.72	-0.30	-1.40	-2.85	2.60	-0.60	0.62	-0.09	-0.27	-2.35	3.32	-0.84	0.57	-0.28	-0.78	-1.24	0.39	-0.29	0.14	-0.03	-2.24	-2.26	-0.14	-0.15	0.01	-0.02
Gompertz	4.14	0.49	6.09	0.22	2.97	0.15	2.12	-0.24	3.44	0.01	0.48	0.03	3.24	0.01	**6.75**	-0.06	1.41	-0.10	0.26	-0.44	0.53	-0.04	0.07	0.00	-1.50	-1.60	-0.16	-0.17	0.11	0.01
Log-logistic	-0.61	-2.67	2.34	-1.11	2.29	-0.37	0.02	-2.03	4.54	-0.36	2.72	-0.03	0.36	-2.09	4.87	-0.80	2.38	-0.31	1.06	-0.52	2.37	-0.02	1.70	0.05	-0.92	-1.25	1.06	0.47	0.87	0.19
Log-normal	-0.25	-2.38	2.56	-0.94	2.07	-0.31	0.99	-1.36	5.62	-0.05	2.93	0.07	1.16	-1.63	5.69	-0.68	2.42	-0.29	1.36	-0.29	2.69	0.16	1.54	0.13	-0.87	-1.18	0.77	0.37	0.46	0.12
Generalized gamma	1.75	-1.06	3.91	-0.37	1.68	0.02	1.20	-0.93	3.74	-0.18	0.48	-0.01	1.79	-0.99	5.45	-0.44	1.50	-0.18	0.97	-0.17	1.03	-0.02	0.11	0.00	-1.68	-1.76	-0.16	-0.16	0.05	0.01
Spline hazard	2.50	-0.13	**3.37**	**-0.08**	0.66	-0.02	1.55	-0.48	3.22	-0.06	0.43	-0.02	1.91	-0.50	4.15	-0.16	0.65	-0.12	0.30	-0.40	0.58	-0.07	0.06	-0.02	-1.55	**-1.62**	-0.12	-0.13	0.00	-0.02
Spline odds	2.58	-0.13	**3.74**	**-0.08**	1.28	-0.03	1.99	-0.29	4.32	0.01	1.32	-0.01	2.07	-0.47	5.02	-0.16	1.53	-0.12	1.35	-0.06	1.63	**0.05**	0.45	0.01	-0.97	-1.20	-0.03	-0.09	0.03	-0.01
Spline normal	2.56	-0.14	3.65	**-0.09**	1.02	-0.03	2.10	-0.22	4.35	0.04	0.98	-0.01	2.20	-0.43	5.07	-0.16	1.22	-0.12	1.09	-0.11	1.29	0.04	0.24	0.00	-1.21	-1.37	-0.09	-0.12	0.01	-0.02

AS, all-cause survival; RS, relative survival; RSMT, restricted mean survival time; CML, chronic myeloid leukemia.

Table B4. Difference in survival proportion at 40 years by cancer site, age group, model, and follow-up time used for extrapolation. The observed values were the Kaplan-Meier survival estimates at lifetime or 40 years. The extrapolated values were retrieved from models fitted to each cohort with 2, 3, 5, and 10 years of follow-up data, and extrapolated to lifetime or 40 years. Difference (%) was calculated as extrapolated minus observed. Bold numbers are values mentioned in the main texts.

	Colon						Breast						Melanoma						Prostate						CML					
Age group	18-59		60-69		70-99		18-59		60-69		70-99		18-59		60-69		70-99		18-59		60-69		70-99		18-59		60-69		70-99	
Survival framework	AS	RS	AS	RS	AS	RS	AS	RS	AS	RS	AS	RS	AS	RS	AS	RS	AS	RS	AS	RS	AS	RS	AS	RS	AS	RS	AS	RS	AS	RS
Survival proportion at lifetime or 40 years (%)	9.19		0.00		0.00		19.31		0.46		0.02		37.95		0.93		0.06		1.62		0.21		0.02		6.73		0.00		0.00	
Extrapolation from 2 years																														
Exponential	-9.18	-9.18	0.00	0.00	0.00	0.00	-1.22	-11.20	12.65	-0.34	0.72	-0.01	-11.03	-22.84	6.00	-0.88	0.20	-0.06	0.73	-1.41	1.25	-0.21	0.04	-0.02	-6.65	-6.69	0.00	0.00	0.00	0.00
Weibull	-9.11	-9.17	0.14	0.00	0.33	0.00	-18.94	-19.22	5.25	-0.41	1.16	-0.01	-26.98	-31.67	0.37	-0.92	-0.05	-0.06	-1.62	-1.62	-0.18	-0.21	0.03	-0.02	-6.73	-6.73	0.00	0.00	0.00	0.00
Gompertz	33.10	1.50	44.90	0.16	45.29	0.00	-19.31	-19.31	-0.46	-0.46	25.53	-0.01	-37.95	-37.95	-0.93	-0.93	-0.06	-0.06	-1.62	-1.62	-0.21	-0.21	-0.01	-0.02	-6.73	-6.73	0.00	0.00	20.17	0.00
Log-logistic	-1.96	-7.35	7.74	0.03	8.21	0.00	-5.76	-14.29	22.99	-0.29	14.31	-0.01	-8.69	-23.02	15.21	-0.86	6.52	-0.06	2.17	-1.41	8.15	-0.20	7.54	-0.02	-3.01	-5.02	4.67	0.02	2.22	0.00
Log-normal	-0.65	-6.99	8.83	0.03	8.42	0.00	15.65	-5.92	42.22	-0.15	22.72	-0.01	13.66	-11.86	30.88	-0.79	10.30	-0.06	8.46	-1.10	17.28	-0.19	11.22	-0.02	-0.30	-3.79	5.87	0.02	1.26	0.00
Generalized gamma	9.72	-4.26	19.52	0.08	21.25	0.00	-19.31	-19.31	-0.46	-0.46	18.74	-0.02	3.05	-16.62	-0.93	-0.93	12.95	-0.06	-1.62	-1.62	-0.21	-0.21	1.49	-0.02	-6.73	-6.73	0.00	0.00	2.45	0.00
Spline hazard	-3.93	-7.71	3.71	0.02	2.04	0.00	-19.28	-19.30	0.66	-0.45	1.34	-0.01	-28.90	-32.85	-0.81	-0.93	-0.05	-0.06	-1.62	-1.62	-0.20	-0.21	0.02	-0.02	-6.66	-6.69	0.00	0.00	0.19	0.00
Spline odds	5.83	-5.30	12.97	0.05	9.69	0.00	-10.73	-16.03	14.53	-0.36	12.73	-0.01	-11.55	-24.53	8.84	-0.89	5.66	-0.06	3.26	-1.31	5.90	-0.20	5.54	-0.02	-0.44	-3.74	1.40	0.00	2.98	0.00
Spline normal	5.37	-5.37	12.15	0.05	8.28	0.00	-2.70	-12.65	23.79	-0.28	14.58	-0.01	2.50	-17.28	14.07	-0.86	5.26	-0.06	4.09	-1.23	6.77	-0.20	4.53	-0.02	-1.44	-4.17	0.38	0.00	1.57	0.00
Extrapolation from 3 years																														
Exponential	-9.15	-9.18	0.02	0.00	0.00	0.00	-3.74	-12.32	11.91	-0.34	0.71	-0.01	-11.79	-23.25	5.91	-0.88	0.25	-0.06	0.13	-1.46	0.98	-0.21	0.04	-0.02	-6.66	-6.69	0.00	0.00	0.00	0.00
Weibull	-8.56	-9.01	0.97	0.01	0.60	0.00	-17.90	-18.77	6.33	-0.39	0.91	-0.01	-22.56	-29.14	1.90	-0.91	0.02	-0.06	-1.62	-1.62	-0.14	-0.21	0.03	-0.02	-6.73	-6.73	0.00	0.00	0.00	0.00
Gompertz	34.85	1.98	**46.00**	0.17	37.13	0.00	-19.31	-19.31	-0.46	-0.46	3.43	-0.01	-37.95	-37.95	-0.93	-0.93	0.09	-0.06	-1.62	-1.62	-0.21	-0.21	0.01	-0.02	-6.73	-6.73	0.00	0.00	10.83	0.00
Log-logistic	0.14	-6.80	10.00	0.04	8.27	0.00	-3.11	-12.90	23.08	-0.28	12.46	-0.01	-5.92	-21.44	17.04	-0.84	7.57	-0.06	3.69	-1.28	8.10	-0.20	6.51	-0.02	-2.60	-4.78	3.94	0.01	1.92	0.00
Log-normal	0.95	-6.55	10.68	0.04	8.02	0.00	12.62	-6.79	37.86	-0.18	17.98	-0.01	11.59	-12.72	28.66	-0.79	9.88	-0.06	7.64	-1.06	13.67	-0.19	8.25	-0.02	-1.40	-4.25	4.13	0.02	0.97	0.00
Generalized gamma	10.55	-3.99	21.12	0.09	14.70	0.00	-14.40	-16.60	9.82	-0.34	4.23	-0.01	2.26	-16.58	12.43	-0.83	8.29	-0.06	-1.01	-1.53	-0.12	-0.21	0.24	-0.02	-6.58	-6.64	0.00	0.00	0.75	0.00
Spline hazard	-0.08	-6.61	11.26	0.06	1.91	0.00	-11.83	-15.95	13.27	-0.31	0.35	-0.01	-9.34	-21.50	6.44	-0.86	0.61	-0.06	-1.48	-1.61	-0.03	-0.21	0.04	-0.02	-6.66	-6.69	0.00	0.00	0.00	0.00
Spline odds	7.90	-4.68	18.36	0.08	8.12	0.00	3.46	-9.82	27.23	-0.23	8.30	-0.01	2.23	-16.65	20.43	-0.82	8.62	-0.06	5.89	-1.09	7.37	-0.20	4.52	-0.02	-1.98	-4.39	1.87	0.01	0.98	0.00
Spline normal	7.15	-4.82	17.60	0.08	6.47	0.00	10.47	-6.78	33.25	-0.19	7.69	-0.01	10.89	-12.41	24.73	-0.79	7.64	-0.06	5.62	-1.07	6.88	-0.20	2.91	-0.02	-3.66	-5.16	0.66	0.00	0.26	0.00
Extrapolation from 5 years																														
Exponential	-8.97	-9.12	0.11	0.00	0.01	0.00	-4.20	-12.47	11.66	-0.34	0.73	-0.01	-9.85	-21.98	7.87	-0.86	0.36	-0.06	-0.10	-1.48	1.00	-0.21	0.04	-0.02	-6.67	-6.70	0.00	0.00	0.00	0.00
Weibull	-5.37	-8.10	3.00	0.02	1.07	0.00	-13.24	-16.53	8.41	-0.36	0.87	-0.01	-11.81	-22.65	8.76	-0.84	0.34	-0.06	-1.54	-1.61	0.16	-0.21	0.03	-0.02	-6.72	-6.73	0.00	0.00	0.00	0.00
Gompertz	35.59	2.29	40.30	0.16	27.73	0.00	-19.27	-19.28	2.60	-0.37	1.34	-0.01	6.50	-11.33	37.57	-0.69	6.35	-0.06	-1.62	-1.62	-0.20	-0.21	0.02	-0.02	-6.73	-6.73	0.00	0.00	4.14	0.00
Log-logistic	3.87	-5.78	11.85	0.05	8.13	0.00	1.98	-10.41	23.34	-0.27	10.55	-0.01	0.63	-17.37	22.75	-0.80	8.15	-0.06	5.24	-1.14	8.71	-0.20	5.10	-0.02	-2.94	-4.93	2.38	0.01	1.54	0.00
Log-normal	4.37	-5.61	12.09	0.05	7.52	0.00	12.09	-6.41	33.20	-0.20	13.27	-0.01	12.15	-11.79	30.31	-0.76	9.00	-0.06	6.95	-1.04	11.44	-0.19	5.33	-0.02	-2.97	-4.98	1.89	0.01	0.67	0.00
Generalized gamma	14.64	-2.84	20.04	0.09	10.03	0.00	1.94	-9.39	15.06	-0.28	1.10	-0.01	10.09	-11.06	27.39	-0.75	6.77	-0.06	2.05	-1.22	2.20	-0.20	0.08	-0.02	-6.42	-6.56	0.00	0.00	0.15	0.00
Spline hazard	11.63	-3.21	11.94	0.07	2.60	0.00	-2.51	-11.37	11.05	-0.32	0.55	-0.01	3.23	-13.69	27.33	-0.71	1.50	-0.06	0.48	-1.39	1.33	-0.20	0.04	-0.02	-6.56	-6.64	0.00	0.00	0.00	0.00
Spline odds	15.46	-2.42	17.36	0.08	7.32	0.00	8.84	-7.06	23.11	-0.25	6.81	-0.01	9.73	-11.53	34.39	-0.71	8.25	-0.06	8.58	-0.82	9.02	-0.19	2.79	-0.02	-3.25	-5.03	0.32	0.00	0.21	0.00
Spline normal	14.98	-2.50	16.42	0.08	5.76	0.00	11.83	-5.71	25.53	-0.23	5.14	-0.01	14.01	-9.66	36.03	-0.70	6.61	-0.06	7.15	-0.89	7.53	-0.19	1.32	-0.02	-4.86	-5.79	0.03	0.00	0.02	0.00
Extrapolation from 10 years																														
Exponential	-7.33	-8.61	0.74	0.01	0.03	0.00	0.69	-10.10	12.73	-0.31	0.69	-0.01	-0.27	**-16.16**	11.68	-0.82	0.51	-0.06	0.47	-1.41	1.09	-0.21	0.03	-0.02	-6.61	-6.67	0.00	0.00	0.00	0.00
Weibull	4.68	-5.26	7.83	0.04	1.18	0.00	2.51	-9.07	12.62	-0.29	0.68	-0.01	8.70	-11.15	16.93	-0.77	0.75	-0.06	-0.23	-1.46	0.70	-0.21	0.01	-0.02	-6.61	-6.66	0.00	0.00	0.00	0.00
Gompertz	33.86	2.37	33.43	0.15	12.43	0.00	25.25	1.11	17.40	-0.16	0.41	-0.01	32.55	-0.40	38.74	-0.66	3.74	-0.06	3.19	-0.94	0.93	-0.20	-0.01	-0.02	-4.71	-5.55	0.00	0.00	0.32	0.00
Log-logistic	10.26	-3.97	14.39	0.06	6.83	0.00	12.05	-5.56	23.80	-0.23	7.48	-0.01	13.64	-9.58	26.57	-0.75	7.13	-0.06	7.24	-0.92	7.77	-0.20	3.32	-0.02	-2.94	-4.87	1.59	0.01	1.21	0.00
Log-normal	10.88	-3.77	14.50	0.07	5.86	0.00	16.82	-3.61	28.73	-0.19	7.93	-0.01	18.49	-7.53	30.39	-0.73	6.80	-0.06	7.50	-0.88	8.29	-0.19	2.75	-0.02	-3.44	-5.13	0.99	0.00	0.43	0.00
Generalized gamma	20.47	-1.16	20.68	0.10	4.47	0.00	18.04	-2.18	18.69	-0.21	0.43	-0.01	22.38	-4.87	29.08	-0.71	3.47	-0.06	5.91	-0.80	2.39	-0.20	0.00	-0.02	-5.60	-6.09	0.00	0.00	0.01	0.00
Spline hazard	24.16	0.84	17.35	0.12	0.97	0.00	18.84	-1.21	15.48	-0.20	0.41	-0.01	22.34	-3.19	21.24	-0.68	0.90	-0.06	2.69	-1.06	1.06	-0.20	-0.01	-0.02	-5.50	-5.99	0.00	0.00	0.00	0.00
Spline odds	24.75	0.85	19.74	0.12	3.43	0.00	22.20	-0.29	22.79	-0.18	3.38	-0.01	23.65	-3.01	27.16	-0.68	4.35	-0.06	8.05	-0.70	5.33	-0.19	0.69	-0.02	-3.05	-4.78	0.13	0.00	0.02	0.00
Spline normal	24.63	0.83	19.15	0.12	2.31	0.00	22.85	-0.01	22.63	-0.18	1.99	-0.01	24.57	-2.82	27.27	-0.68	2.91	-0.06	6.54	-0.78	3.70	-0.20	0.19	-0.02	-4.16	-5.31	0.02	0.00	0.00	0

AS, all-cause survival; RS, relative survival; RSMT, restricted mean survival time; ; CML, chronic myeloid leukemia.
