# Supplementary material for: Comparing Survival Extrapolation within All-Cause and Relative Survival Frameworks by Standard Parametric Models and Flexible Parametric Spline Models Using the Swedish Cancer Registry
Source: Med Decis Making. 2024 Feb 5;44(3):269–82. doi: 10.1177/0272989X241227230 (PMC10988990; doi:10.1177/0272989X241227230)

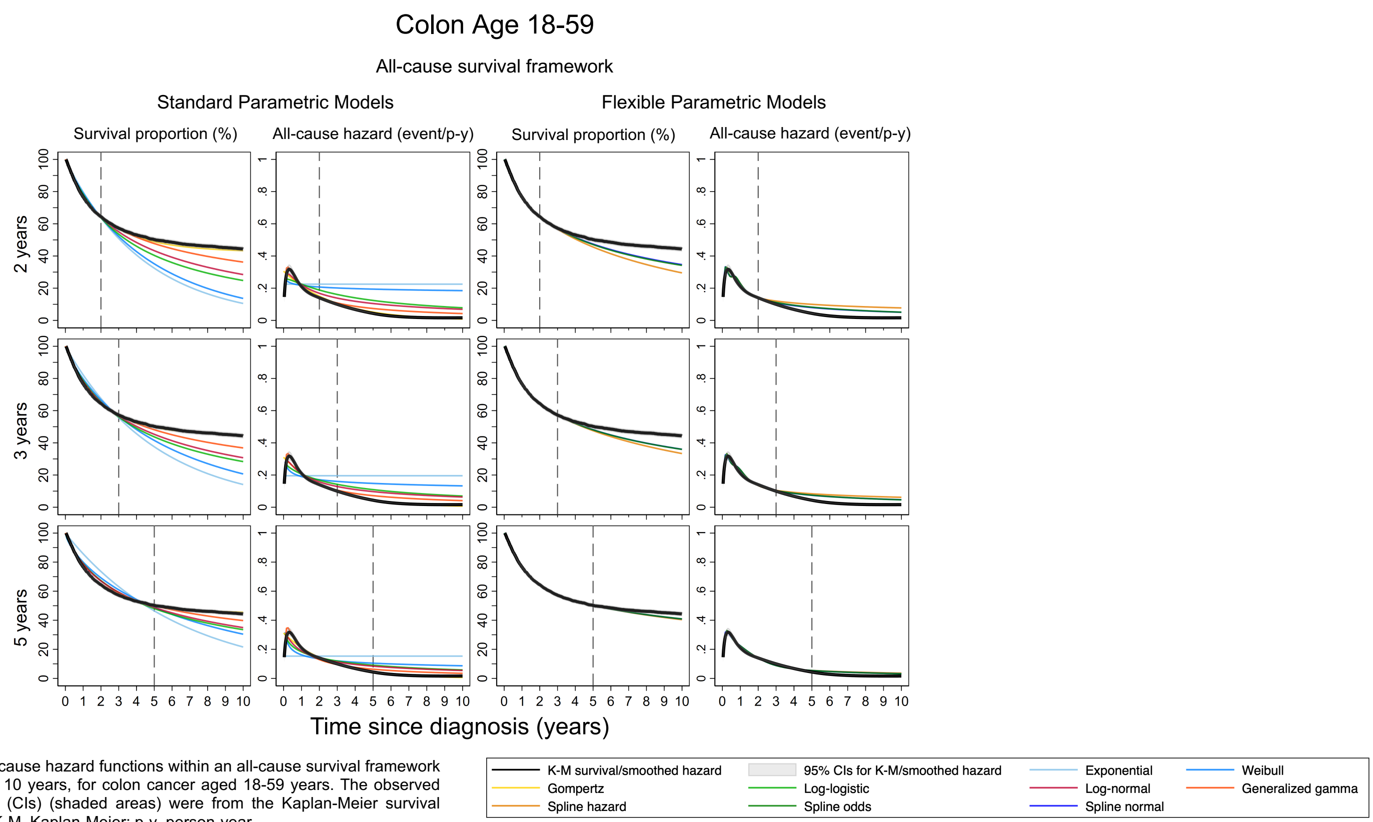

Figure C1. Plots show the extrapolated survival and all-cause hazard functions within an all-cause survival framework by model, and follow-up time used for extrapolation to 10 years, for colon cancer aged 18-59 years. The observed estimates (black lines) with 95% confidence intervals (CIs) (shaded areas) were from the Kaplan-Meier survival estimates or the smoothed all-cause hazard functions. K-M, Kaplan-Meier; p-y, person-year.

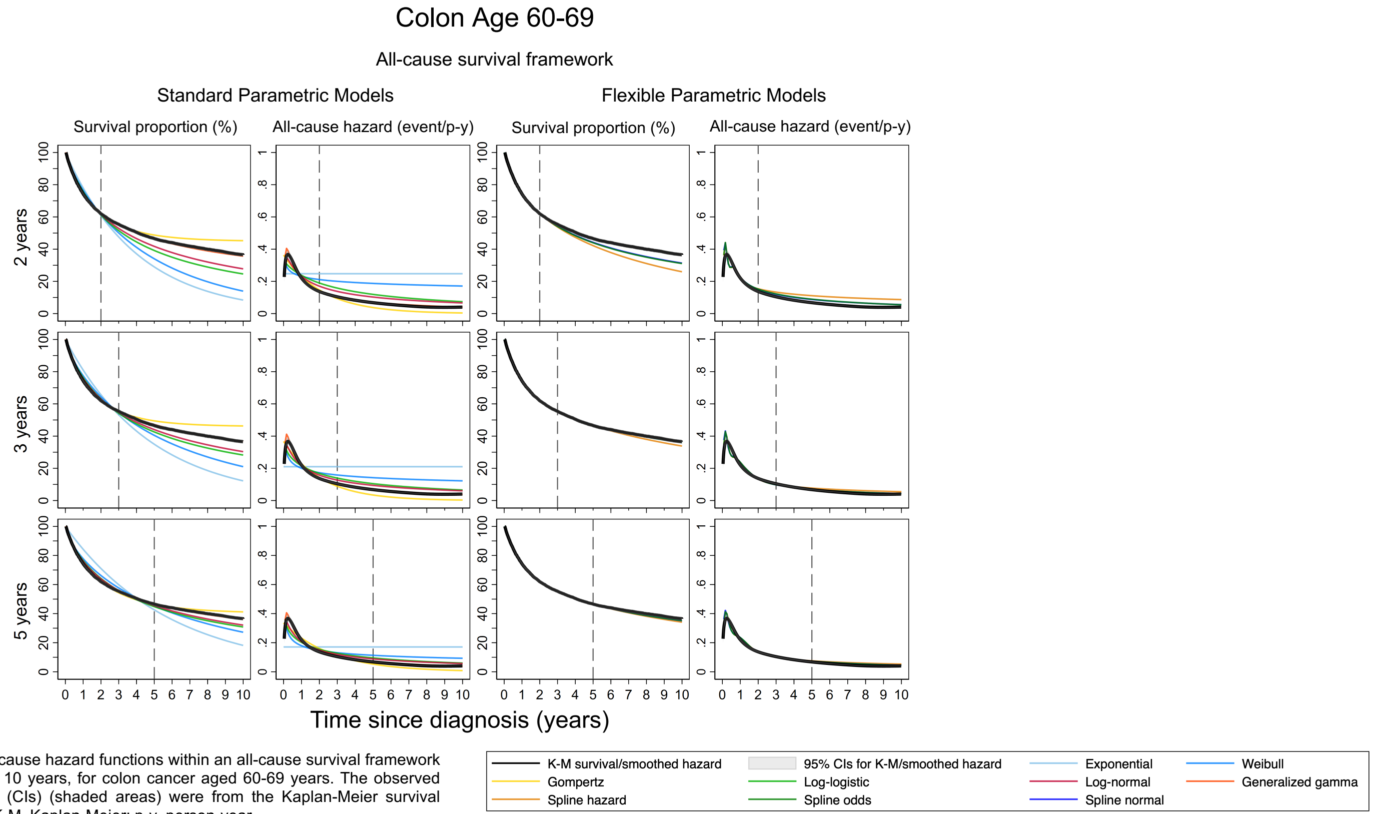

Figure C2. Plots show the extrapolated survival and all-cause hazard functions within an all-cause survival framework by model, and follow-up time used for extrapolation to 10 years, for colon cancer aged 60-69 years. The observed estimates (black lines) with 95% confidence intervals (CIs) (shaded areas) were from the Kaplan-Meier survival estimates or the smoothed all-cause hazard functions. K-M, Kaplan-Meier; p-y, person-year.

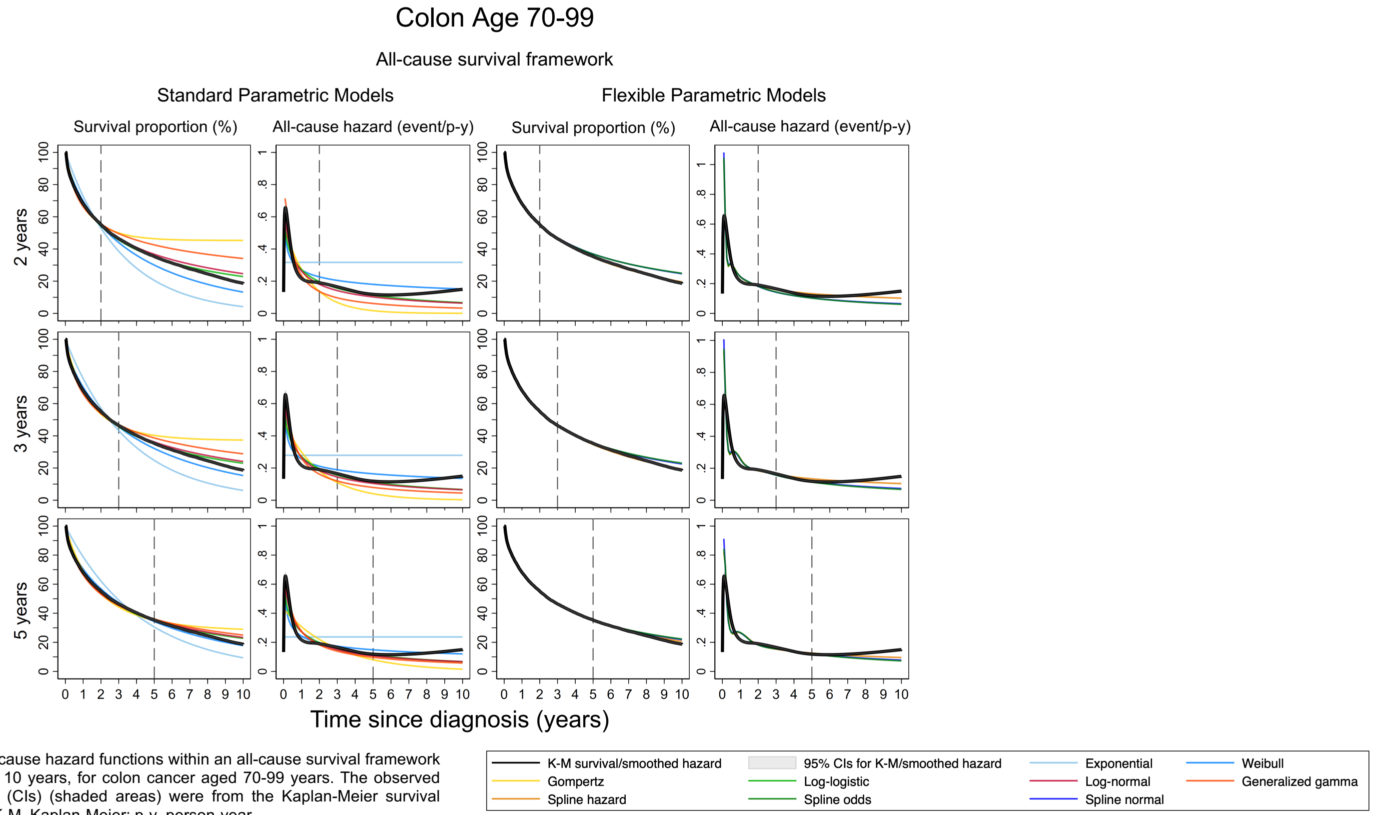

Figure C3. Plots show the extrapolated survival and all-cause hazard functions within an all-cause survival framework by model, and follow-up time used for extrapolation to 10 years, for colon cancer aged 70-99 years. The observed estimates (black lines) with 95% confidence intervals (CIs) (shaded areas) were from the Kaplan-Meier survival estimates or the smoothed all-cause hazard functions. K-M, Kaplan-Meier; p-y, person-year.

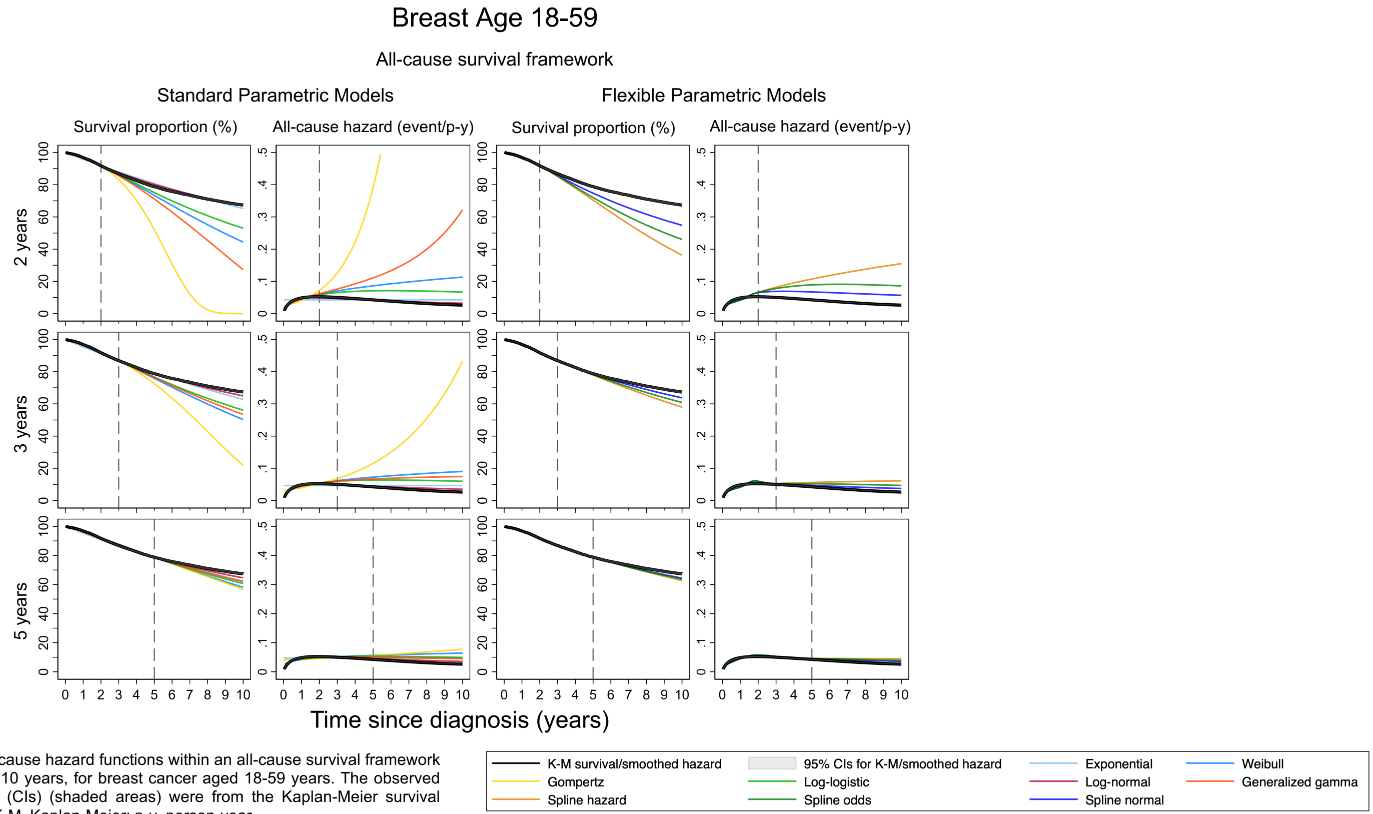

Figure C4. Plots show the extrapolated survival and all-cause hazard functions within an all-cause survival framework by model, and follow-up time used for extrapolation to 10 years, for breast cancer aged 18-59 years. The observed estimates (black lines) with 95% confidence intervals (CIs) (shaded areas) were from the Kaplan-Meier survival estimates or the smoothed all-cause hazard functions. K-M, Kaplan-Meier; p-y, person-year.

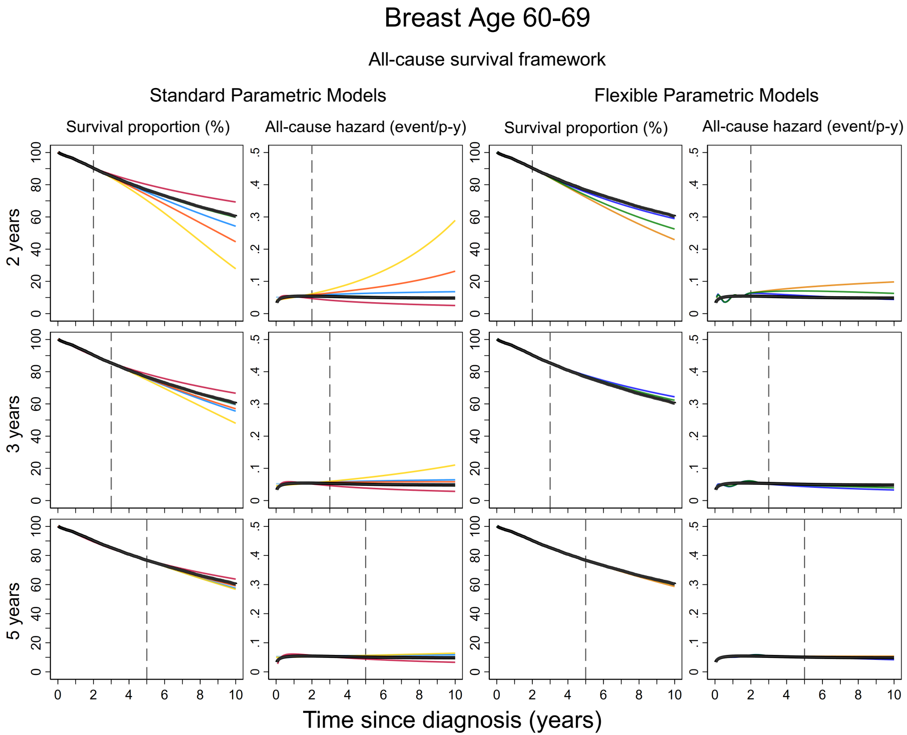

Figure C5. Plots show the extrapolated survival and all-cause hazard functions within an all-cause survival framework by model, and follow-up time used for extrapolation to 10 years, for breast cancer aged 60-69 years. The observed estimates (black lines) with 95% confidence intervals (CIs) (shaded areas) were from the Kaplan-Meier survival estimates or the smoothed all-cause hazard functions. K-M, Kaplan-Meier; p-y, person-year.

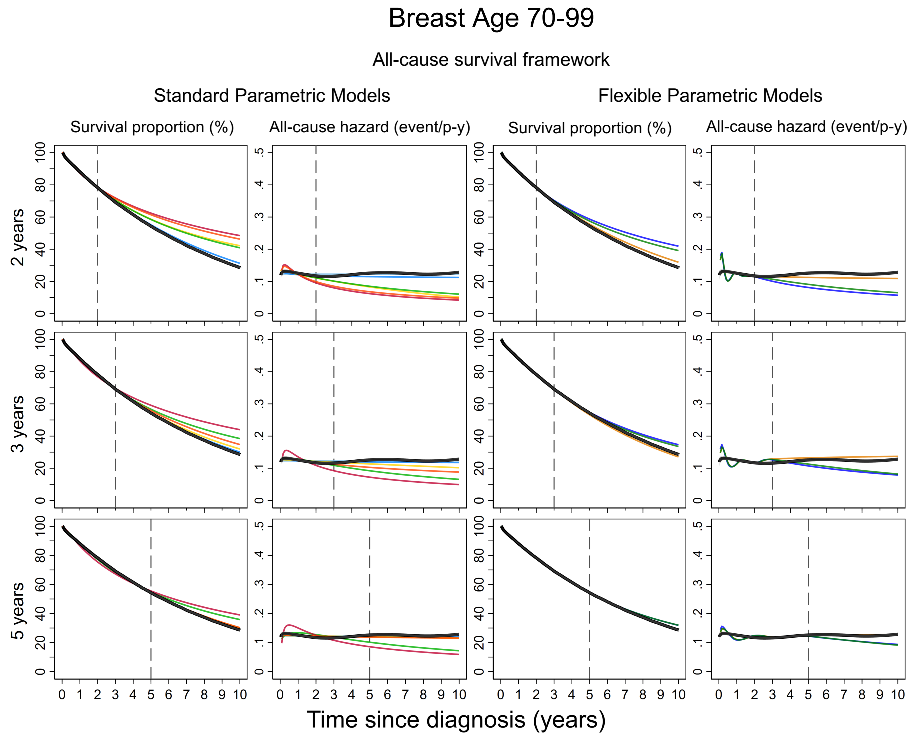

Figure C6. Plots show the extrapolated survival and all-cause hazard functions within an all-cause survival framework by model, and follow-up time used for extrapolation to 10 years, for breast cancer aged 70-99 years. The observed estimates (black lines) with 95% confidence intervals (CIs) (shaded areas) were from the Kaplan-Meier survival estimates or the smoothed all-cause hazard functions. K-M, Kaplan-Meier; p-y, person-year.

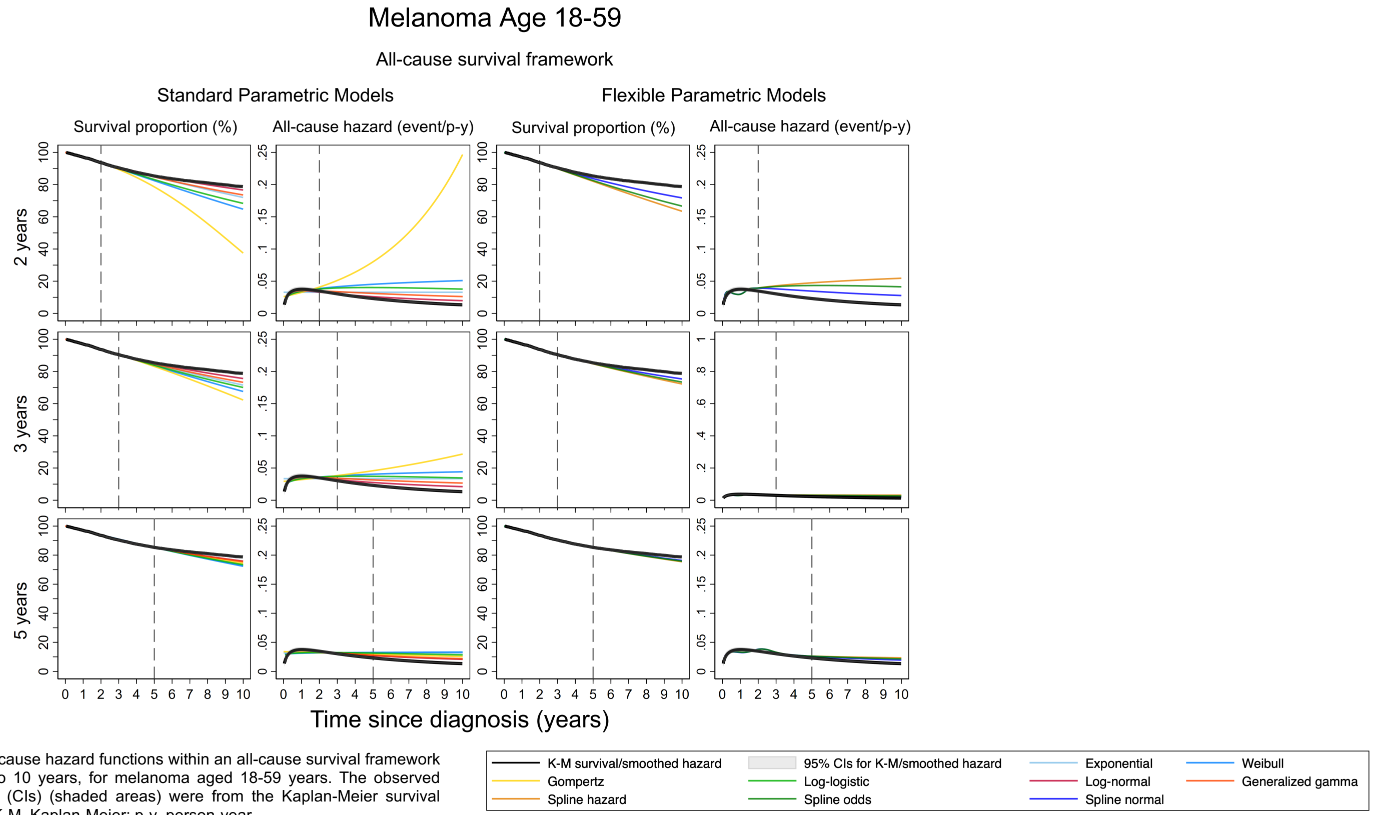

Figure C7. Plots show the extrapolated survival and all-cause hazard functions within an all-cause survival framework by model, and follow-up time used for extrapolation to 10 years, for melanoma aged 18-59 years. The observed estimates (black lines) with 95% confidence intervals (CIs) (shaded areas) were from the Kaplan-Meier survival estimates or the smoothed all-cause hazard functions. K-M, Kaplan-Meier; p-y, person-year.

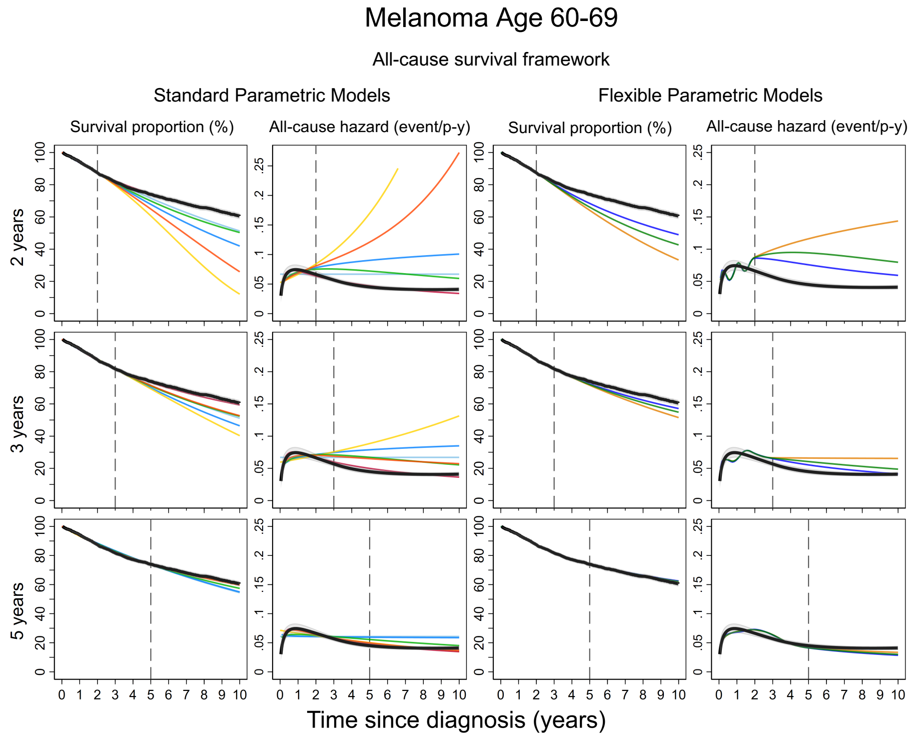

Figure C8. Plots show the extrapolated survival and all-cause hazard functions within an all-cause survival framework by model, and follow-up time used for extrapolation to 10 years, for melanoma aged 60-69 years. The observed estimates (black lines) with 95% confidence intervals (CIs) (shaded areas) were from the Kaplan-Meier survival estimates or the smoothed all-cause hazard functions. K-M, Kaplan-Meier; p-y, person-year.

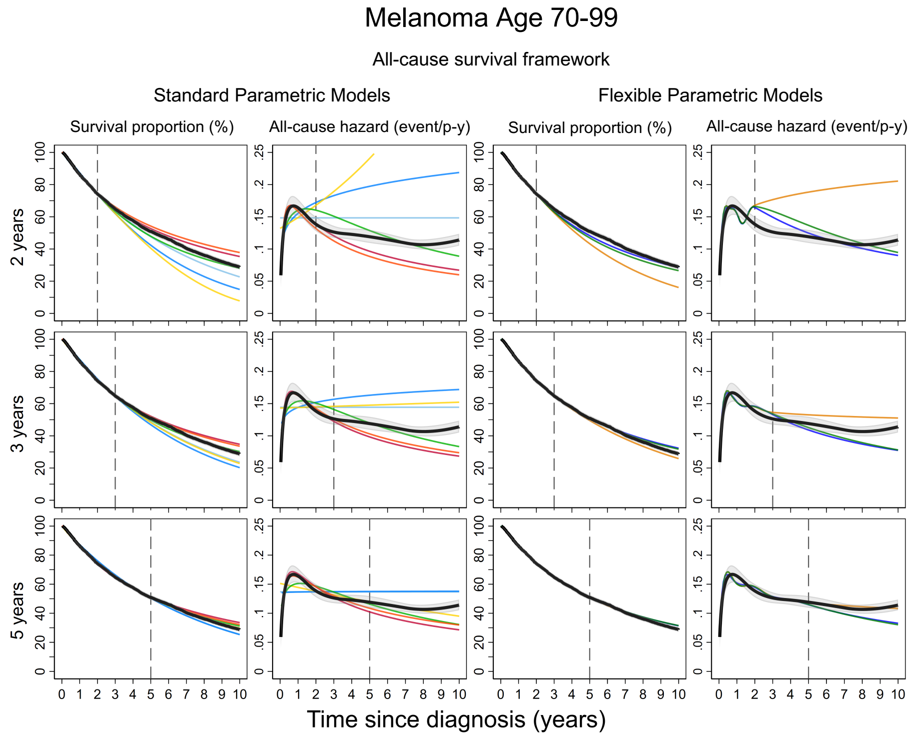

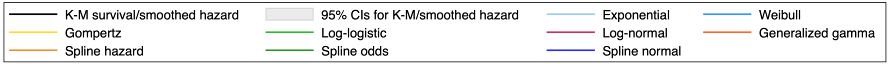

Figure C9. Plots show the extrapolated survival and all-cause hazard functions within an all-cause survival framework by model, and follow-up time used for extrapolation to 10 years, for melanoma aged 70-99 years. The observed estimates (black lines) with 95% confidence intervals (CIs) (shaded areas) were from the Kaplan-Meier survival estimates or the smoothed all-cause hazard functions. K-M, Kaplan-Meier; p-y, person-year.

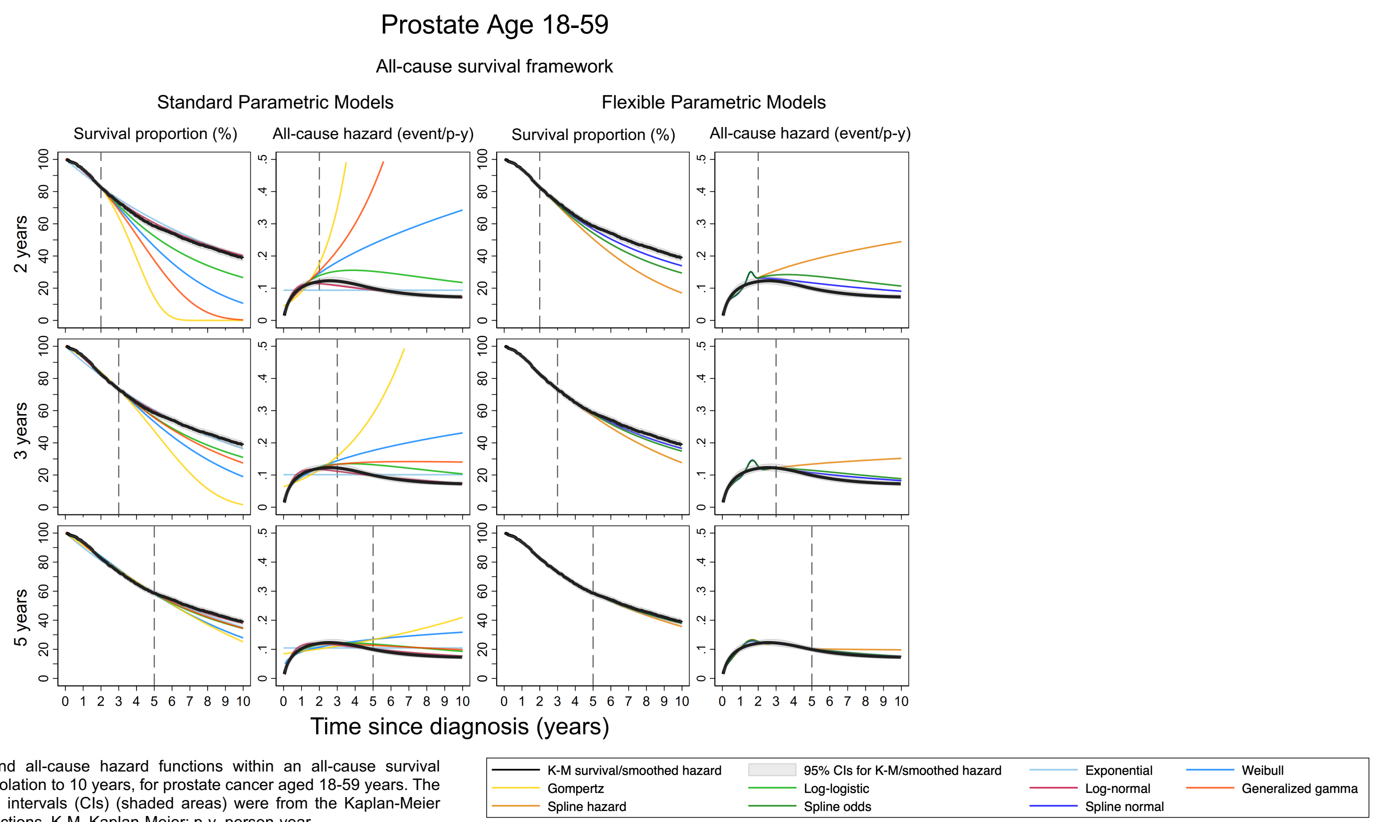

Figure C10. Plots show the extrapolated survival and all-cause hazard functions within an all-cause survival framework by model, and follow-up time used for extrapolation to 10 years, for prostate cancer aged 18-59 years. The observed estimates (black lines) with 95% confidence intervals (CIs) (shaded areas) were from the Kaplan-Meier survival estimates or the smoothed all-cause hazard functions. K-M, Kaplan-Meier; p-y, person-year.

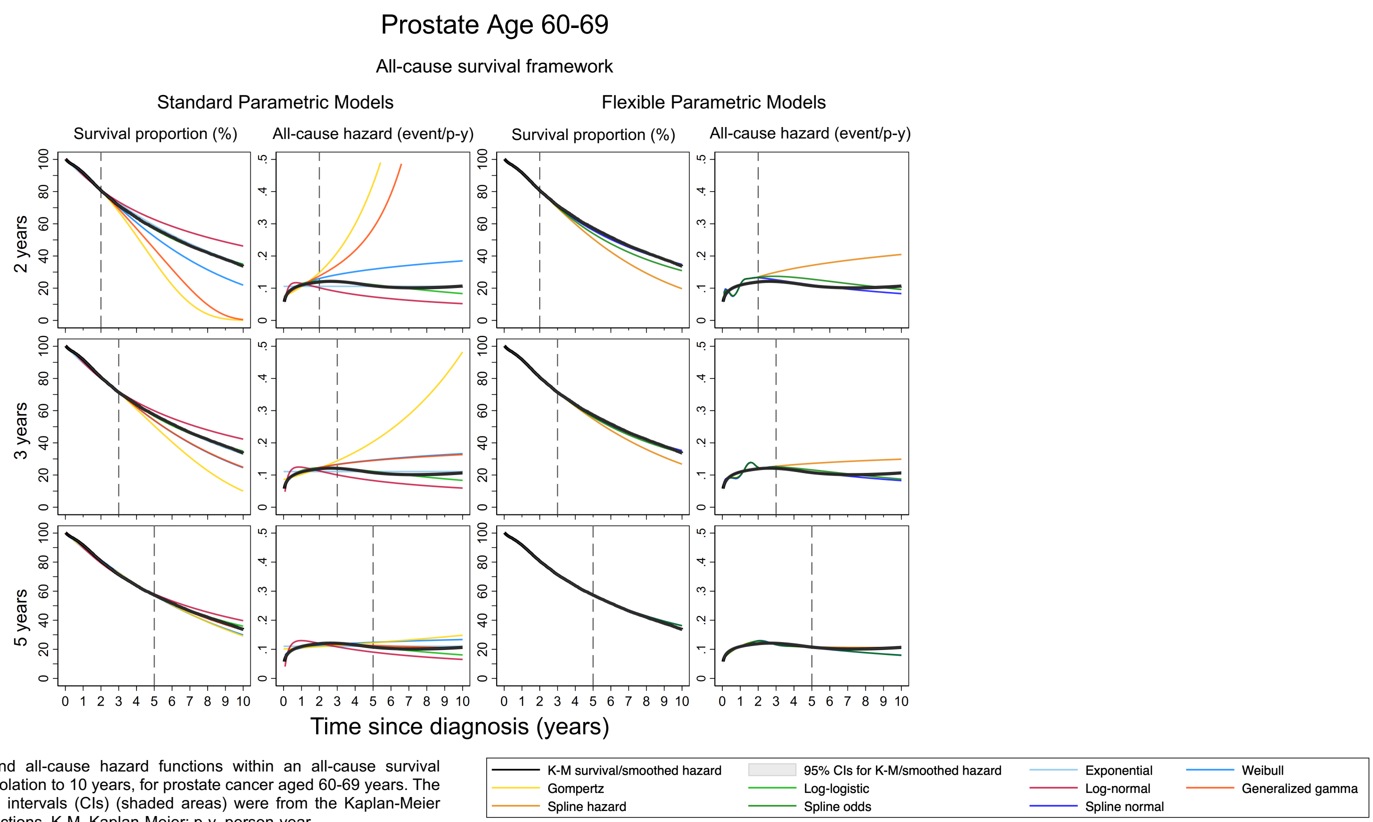

Figure C11. Plots show the extrapolated survival and all-cause hazard functions within an all-cause survival framework by model, and follow-up time used for extrapolation to 10 years, for prostate cancer aged 60-69 years. The observed estimates (black lines) with 95% confidence intervals (CIs) (shaded areas) were from the Kaplan-Meier survival estimates or the smoothed all-cause hazard functions. K-M, Kaplan-Meier; p-y, person-year.

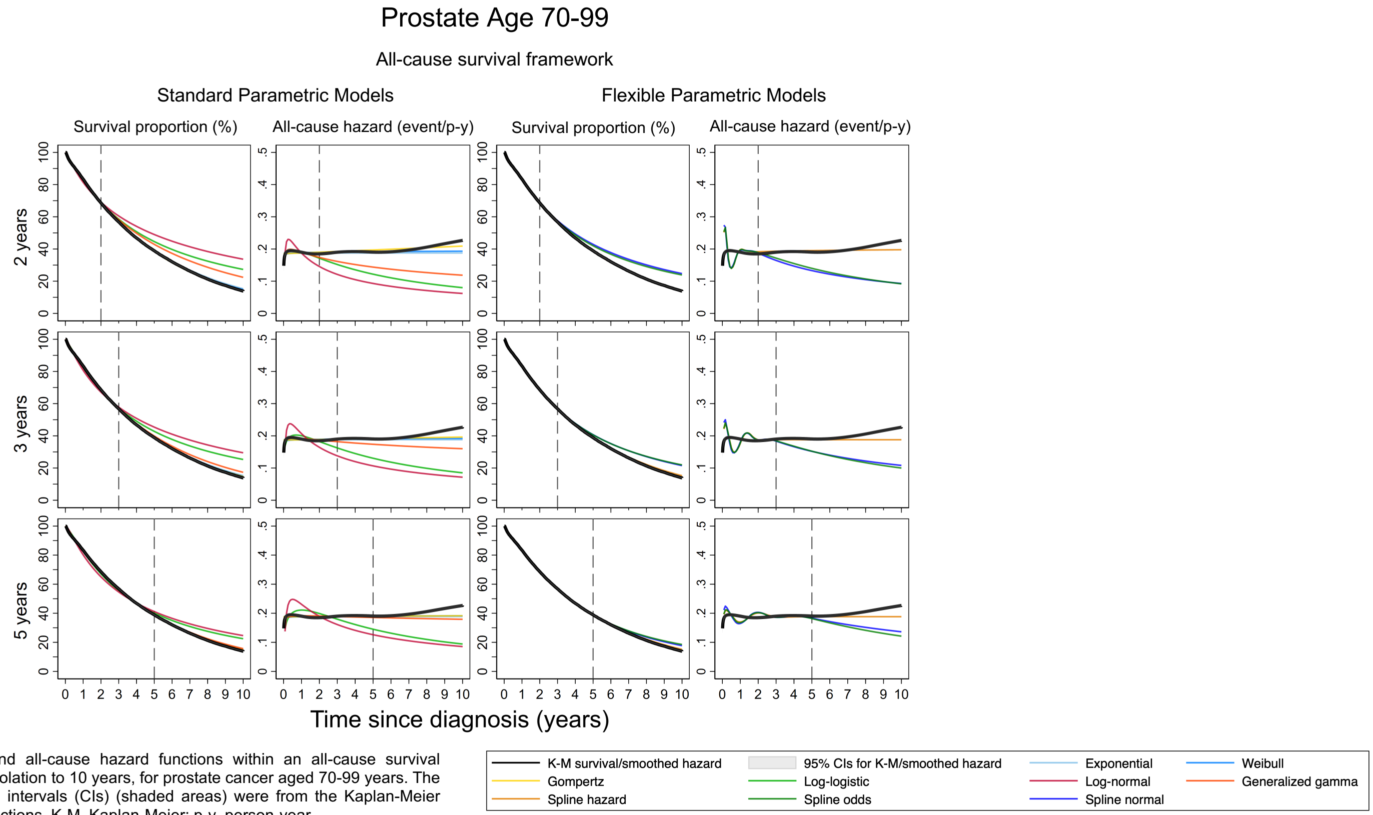

Figure C12. Plots show the extrapolated survival and all-cause hazard functions within an all-cause survival framework by model, and follow-up time used for extrapolation to 10 years, for prostate cancer aged 70-99 years. The observed estimates (black lines) with 95% confidence intervals (CIs) (shaded areas) were from the Kaplan-Meier survival estimates or the smoothed all-cause hazard functions. K-M, Kaplan-Meier; p-y, person-year.

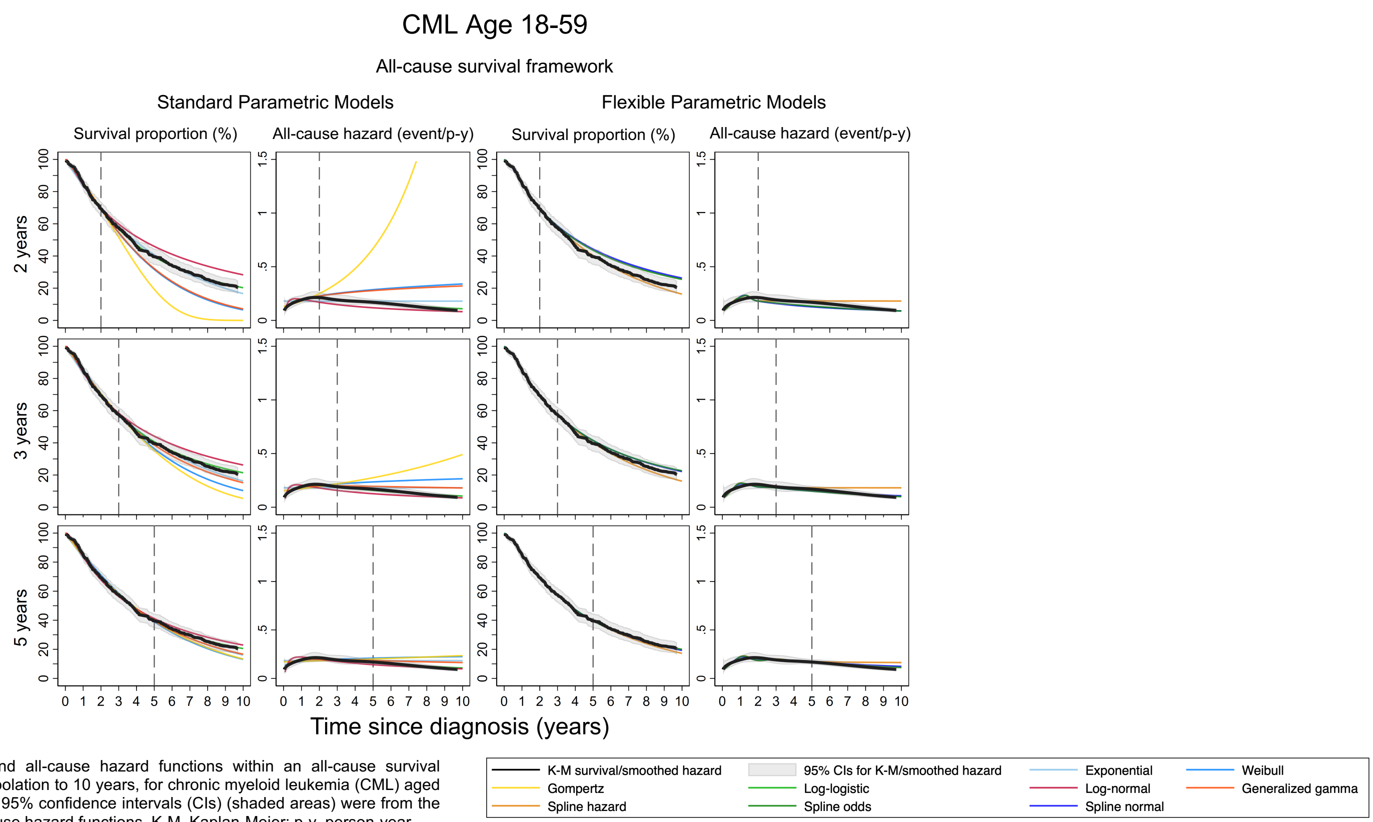

Figure C13. Plots show the extrapolated survival and all-cause hazard functions within an all-cause survival framework by model, and follow-up time used for extrapolation to 10 years, for chronic myeloid leukemia (CML) aged 18-59 years. The observed estimates (black lines) with 95% confidence intervals (CIs) (shaded areas) were from the Kaplan-Meier survival estimates or the smoothed all-cause hazard functions. K-M, Kaplan-Meier; p-y, person-year.

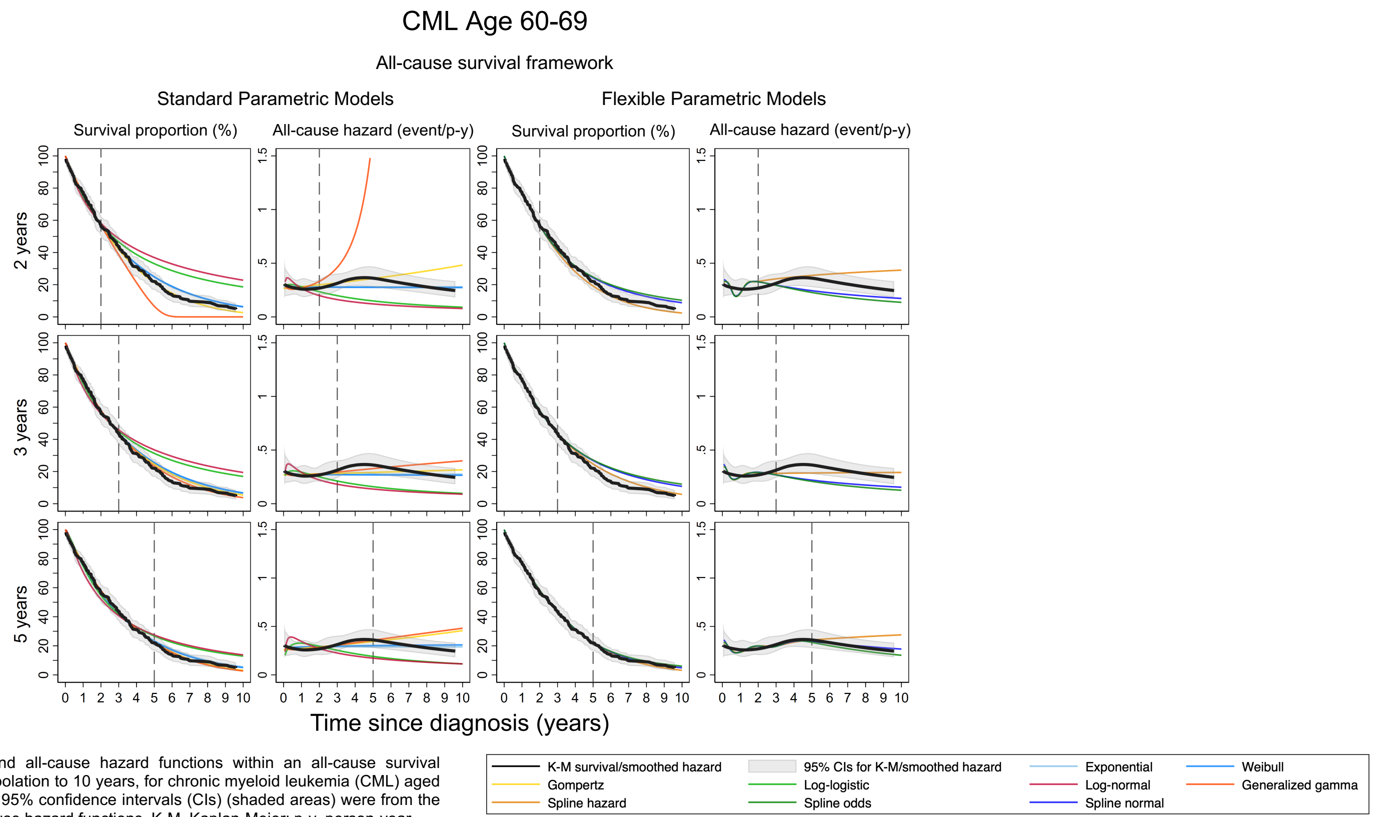

Figure C14. Plots show the extrapolated survival and all-cause hazard functions within an all-cause survival framework by model, and follow-up time used for extrapolation to 10 years, for chronic myeloid leukemia (CML) aged 60-69 years. The observed estimates (black lines) with 95% confidence intervals (CIs) (shaded areas) were from the Kaplan-Meier survival estimates or the smoothed all-cause hazard functions. K-M, Kaplan-Meier; p-y, person-year.

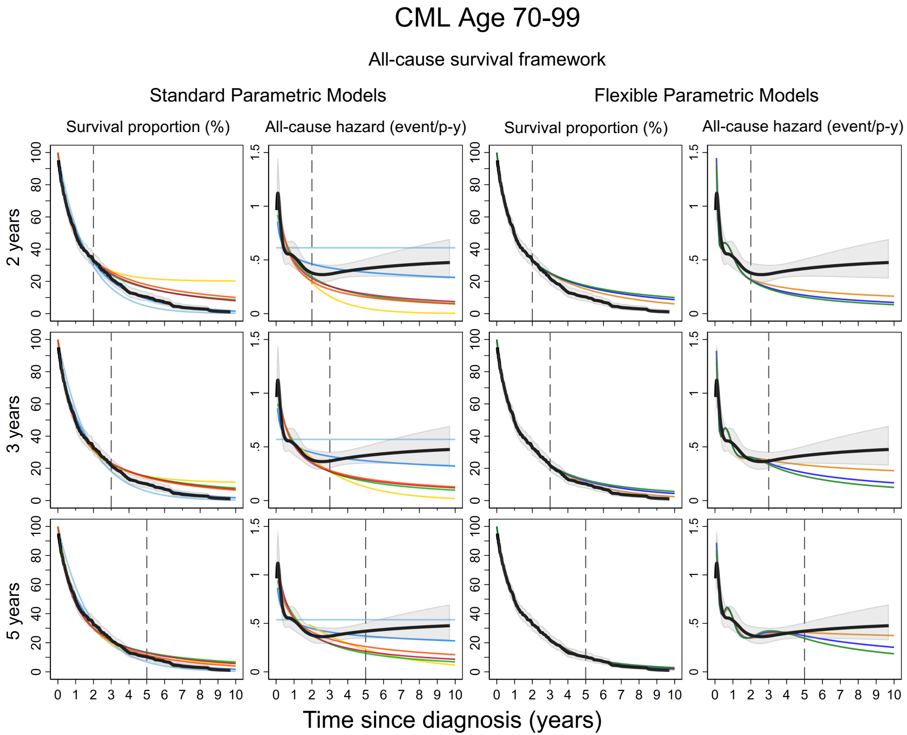

Figure C15. Plots show the extrapolated survival and all-cause hazard functions within an all-cause survival framework by model, and follow-up time used for extrapolation to 10 years, for chronic myeloid leukemia (CML) aged 70-99 years. The observed estimates (black lines) with 95% confidence intervals (CIs) (shaded areas) were from the Kaplan-Meier survival estimates or the smoothed all-cause hazard functions. K-M, Kaplan-Meier; p-y, person-year.

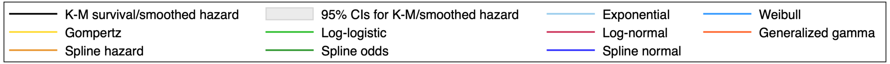

Supplement: sj-pdf-3-mdm-10.1177_0272989X241227230 – Supplemental material for Comparing Survival Extrapolation within All-Cause and Relative Survival Frameworks by Standard Parametric Models and Flexible Parametric Spline Models Using the Swedish Cancer Registry [file sj-pdf-3-mdm-10.1177_0272989X241227230.pdf]
